# Quartz crystal microbalances (QCM) are suitable for real-time dosimetry in nanotoxicological studies using VITROCELL®Cloud cell exposure systems

**DOI:** 10.1186/s12989-020-00376-w

**Published:** 2020-09-16

**Authors:** Yaobo Ding, Patrick Weindl, Anke-Gabriele Lenz, Paula Mayer, Tobias Krebs, Otmar Schmid

**Affiliations:** 1 Institute of Lung Biology and Disease, Helmholtz Zentrum München, 85764 Neuherberg, Germany; 2 Comprehensive Pneumology Center, Munich (CPC-M) - Member of the German Center for Lung Research (DZL), 81377 Munich, Germany; 3 VITROCELL Systems GmbH, 79183 Waldkirch, Germany

**Keywords:** Quartz crystal microbalance, Dosimetry, Air-liquid interface cell exposure, Aerosol exposure, Nanotoxicology, Cloud, Vitrocell, Nanoparticles

## Abstract

**Background:**

Accurate knowledge of cell−/tissue-delivered dose plays a pivotal role in inhalation toxicology studies, since it is the key parameter for hazard assessment and translation of in vitro to in vivo dose-response. Traditionally, (nano-)particle toxicological studies with in vivo and in vitro models of the lung rely on *in silio* computational or off-line analytical methods for dosimetry. In contrast to traditional in vitro testing under submerged cell culture conditions, the more physiologic air-liquid interface (ALI) conditions offer the possibility for real-time dosimetry using quartz crystal microbalances (QCMs). However, it is unclear, if QCMs are sensitive enough for nanotoxicological studies. We investigated this issue for two commercially available VITROCELL®Cloud ALI exposure systems.

**Results:**

Quantitative fluorescence spectroscopy of fluorescein-spiked saline aerosol was used to determine detection limit, precision and accuracy of the QCMs implemented in a VITROCELL®Cloud 6 and Cloud 12 system for dose-controlled ALI aerosol-cell exposure experiments. Both QCMs performed linearly over the entire investigated dose range (200 to 12,000 ng/cm^2^) with an accuracy of 3.4% (Cloud 6) and 3.8% (Cloud 12). Their precision (repeatability) decreased from 2.5% for large doses (> 9500 ng/cm^2^) to values of 10% and even 25% for doses of 1000 ng/cm^2^ and 200 ng/cm^2^, respectively. Their lower detection limit was 170 ng/cm^2^ and 169 ng/cm^2^ for the Cloud 6 and Cloud 12, respectively. Dose-response measurements with (NM110) ZnO nanoparticles revealed an onset dose of 3.3 μg/cm^2^ (or 0.39 cm^2^/cm^2^) for both cell viability (WST-1) and cytotoxicity (LDH) of A549 lung epithelial cells.

**Conclusions:**

The QCMs of the Cloud 6 and Cloud 12 systems show similar performance and are highly sensitive, accurate devices for (quasi-) real-time dosimetry of the cell-delivered particle dose in ALI cell exposure experiments, if operated according to manufacturer specifications. Comparison with in vitro onset doses from this and previously published ALI studies revealed that the detection limit of 170 ng/cm^2^ is sufficient for determination of toxicological onset doses for all particle types with low (e.g. polystyrene) or high mass-specific toxicity (e.g. ZnO and Ag) investigated here. Hence, in principle QCMs are suitable for in vitro nanotoxciological studies, but this should be investigated for each QCM and ALI exposure system under the specific exposure conditions as described in the present study.

## Background

Nanotechnology and nanomaterials such as engineered nanoparticles have found wide-spread application in everyday life products, such as cosmetics, food and clothing [1–3]. In addition, nanomaterials are often used as intermediate products during manufacturing processes, for example as catalysts [4] and additives [5]. It has been found that release of nanoparticles into the environment can occur during daily uses of consumer products as well as during production processes [6], which can result in inhalation exposure of customers or workers with nanoparticles at home or at workplaces [7]. Therefore, investigation of adverse effects of nanoparticles on human health has received considerable attention. Toxicological studies have shown that in-vitro cell exposure to nanoparticles can trigger inflammatory responses or cell death, if elevated dose levels are given [8–12]. In-vivo animal studies revealed both short-term toxic and long-term health effects including inflammation, cancer and fibrosis [13]. Moreover, nanoparticles are capable of being translocated across the air-blood barrier of the lungs via blood circulation into secondary organs, such as brain, liver and kidney, causing further toxicities to those parts of human body [14, 15].

One of the major difficulties nowadays for hazard and risk assessment of nanoparticles and fibers is accurate dosimetry. Often, toxicological studies provide precise information on nanoparticle concentration at exposure site in the unit of mass per air volume (μg/m^3^) or mass per cell medium volume (μg/ml) for in vitro submerged exposures. However, the true delivered dose to the cells is largely overlooked and thus remains unknown, especially in the cases of in vitro cell exposure methods [16, 17].

Traditionally, in-vitro nanoparticle-cell exposure experiments are performed under submerged culture conditions, i.e. cells are covered completely with cell culture medium and the nanoparticles are added directly into the cell culture medium. While this method maybe more time- and cost-effective, it has some severe drawbacks for inhalation toxicology studies. Firstly, by adding the nanoparticles directly into the cell culture medium, the particles may adsorb proteins contained in the medium onto their surfaces resulting into a protein corona, which has been shown to alter particle toxicity [18–20]. Secondly, the cell-delivered dose, which is the toxicology relevant dose that governs dose response relationships, remains difficult to determine under submerged conditions. The amount of particles that reach the cells located at the bottom of the multi-well plate is governed by particle kinetics due to diffusion and sedimentation, which depends on both particle characteristics (e.g., size, shape, degree of agglomeration, density) and medium properties (viscosity, density) [21]. Advanced numerical models have been developed to help researchers to tackle this problem [22, 23], however uncertainties remain mainly due to measurement uncertainties in the input parameters (e.g. DLS size distribution, effective particle density) variability in these parameters during the incubation period e.g. in size distribution due to particle agglomeration and/or faster deposition of larger agglomerates. Moreover, application of these dosimetric tools requires availability of various devices (particle sizer, ultracentrifuge and particle volume measurement cuvette), some experience with operating Matlab software codes and sometimes a high level of expertise and good understandings of the different physical processes involved in particokinetics [24].

Alternatively, air-liquid interface (ALI) cell exposures are physiologically more relevant representations of cellular exposures to inhaled airborne particles. In addition, they allow in some cases for real-time dosimetry of the cell-delivered particles dose. In ALI exposures, nanoparticle aerosol is deposited directly onto the cells cultured at the air-liquid interface (air on the apical and medium on the basal side of the cells). This not only mimics the real-life scenario in the lung during particle inhalation, it also avoids nanoparticle-medium artefacts (protein corona, agglomeration) and allows pulmonary epithelial cells to polarize and secrete protective liquid lining fluids resembling physiologic conditions. Different ALI exposure systems have been developed, which can be categorized based on their distinct aerosol delivery mechanisms, such as diffusion and sedimentation [25], thermophoresis [26], electrophoresis [27, 28] and cloud dynamics [29, 30]. Moreover, the cell-delivered particle dose can be measured by standard analytical techniques such as radio−/fluorospectrometry, Scanning or Transmission Electron Microscopy (SEM or TEM), high-pressure liquid chromatography (HPLC), and Inductively Coupled Plasma Mass Spectrometry (ICP-MS). For this, the aerosol dose deposited onto the membrane of the transwell inserts where cells are cultured under air-liquid interface conditions is quantitatively determined either in situ with SEM [25], TEM [31] or fluorescence microscopy [26] or ex situ after sample collection with HPLC [32], ICP-MS [33], atomic absorption spectroscopy [30, 34–40] or trace-based radio−/fluorospectrometry [29, 41] or tracer-free chemical fluorospectrometry [42]. All of the above methods only allow for off-line dosimetry. For real-time dosimetry, mostly indirect methods were used where the change in aerosol concentration downstream of the transwell inserts was assessed with and without transwell inserts in place using standard aerosol measurement technologies such as cascade impactors [43], electrical low pressure impactors (ELPI) or electrical mobility classification combined with condensation particle counters [44]. Ideally, these real-time approaches should be validated with an off-line dosimetry method. The methods listed above often require complex sample extraction, expensive analytical equipment and/or elaborate data analysis efforts. Moreover, many of these analytical methods are restricted to certain material types or the use of tracers and they are not suitable for real-time dosimetry. Hence, real-time measurement methods for direct monitoring of the cell-delivered aerosol dose, which are accurate and sensitive enough for air-liquid interface cell culture experiments, could greatly facilitate dose-controlled toxicity studies with aerosolized nanoparticles. Quartz crystal microbalances have been suggested to fill this void [30]. The QCM is a device that measures the mass deposited onto a resonating quartz crystal (surrogate for cell layer) by detecting the change in eigenfrequency of this quartz crystal. Thus, it utilizes the principle of a “tuning fork” as the eigenfrequency (frequency of sound) of a tuning fork decreases (lowers) with increasing mass of the tuning fork. Since its first description by Raleigh (1885), the QCM has found numerous applications in modern science and technology including gas phase detector, immunosensor and DNA biosensor [45–50]. Due to its high sensitivity the QCM can be also used in nanotoxicological studies, in which the applied nanoparticle mass dose is often very low, especially for highly toxic materials. In addition, thanks to their unique measurement principle, QCMs are generally material-independent and can be used to measure different types of particles. However, there is some controversy in the nanotoxicology community as to whether QCMs are sensitive and accurate enough for toxicological dose-response measurements with aerosolized nanoparticles using air-liquid interface aerosol-cell exposure systems. This issue will be addressed here.

As for any real-time measurement device QCM stability over the entire measurement period is essential for reliable dose measurement. The dose rate for aerosol-cell delivery and, hence, the required exposure time for reaching the onset dose varies by several orders of magnitude depending on the type of exposure system [51]. In general low dose rates and hence long exposure times are more difficult to monitor with QCMs due to limited zero-point stability and cross-sensitivity to other potentially varying parameters such as temperature, relative humidity and vibration of the cell exposure and hence QCM system [52, 53]. For minimization of these adverse effects we employ the VITROCELL® Cloud system, the commercial version of the ALICE Cloud technology introduced by us a few years ago [29], which allows for delivery of high amounts of dose within a few minutes for almost all inhalable, non-volatile (at 37 °C) and solid particles or (water-)soluble chemicals as compared to other ALI cell exposure systems. For instance 100-fold to 1000-fold higher dose rates have been reported as compared to standard stagnation point aerosol-cell exposure systems with or without electrostatic deposition enhancement [51].

In this study, we characterize the performance of the QCMs integrated in two types of the VITROCELL® Cloud system (Cloud 6 and 12) for air-liquid interface cell exposure. We experimentally determine accuracy, precision and sensitivity (lower limit of detection) of the QCMs. Moreover, we report on cytotoxicity (WST1, LDH) dose-response measurements with human epithelial cells (A549) for zinc oxide (ZnO) nanoparticles, a known highly toxic material in inhalation toxicology verifying the derived detect limit of the QCM. The observed onset dose for ZnO nanoparticles is put into perspective with previously reported toxicological onset doses reported for different types of nanoparticles obtained with air-liquid interface cell exposure systems. This demonstrates the suitability of the VITROCELL® Cloud QCMs for in vitro toxicity studies at the air-liquid interface and it provides guidance under which experimental conditions QCMs are useful dosimetry tools for nanoparticle toxicity studies.

## Methods

### Quartz crystal microbalance (QCM)

A QCM consists of a piezo-electrically driven quartz crystal which is kept vibrating at its resonance frequency (eigenfrequency) by an electric feedback oscillation circuit [54]. Upon applying a voltage the piezoactive crystal will change its geometry (mechanical deformation) and subsequently turn back to its original state within a certain period of time (relaxation). Hence, applying an alternating voltage to the crystal at its eigenfrequency induces oscillations with maximum amplitude.

The eigenfrequency of the quartz crystal is influenced by various factors such as its shape, thickness, mass and crystalline structure. In the case of a QCM, any additional mass deposited onto the crystal’s surface can be considered as an increase of the crystal’s mass, which in turn decreases the resonance frequency (eigenfrequency) of the oscillator, analogous to a tuning fork, which also resonates at a low (eigen-)frequency (pitch), if its mass is increased. The change in eigenfrequency corresponds to the mass deposited on the crystal, and this can be measured by using a standard precision frequency counter. This mass detection method was discovered by the German physicist Günter Sauerbrey in 1959 who also described the governing equation relating the changes in mass and eigenfrequency [55]:

1 $$ \varDelta f=-\frac{2{f}_0^2}{A\sqrt{\rho_q{\mu}_q}}\varDelta m $$

$$ {f}_0: Eigenfrequency\ of\ quartz\ crystal $$

$$ \Delta  f: Change\ in\ eigenfrequency\  due\  to\ the\ mass\ deposited\ onto\ the\ quartz\ crystal $$

$$ A: Active\ crystal\ area, $$

$$ {\rho}_q: Density\ of\ quartz. $$

$$ {\mu}_q: Shear\ modulus\ of\ quartz $$

$$ \varDelta m: Mass\ deposited\  on\  quartz\ crystal $$

The QCMs incorporated in the VITROCELL® Cloud systems have an eigenfrequency of 5 MHz. The diameter and resistance are 14 mm and ~ 10 Ohm for the VITROCELL® Cloud 6 and 1 in. (=25.4 mm) and < 15 Ohm for the VITROCELL® Cloud 12, respectively (VITROCELL Systems, Waldkirch, Germany).

The application of Sauerbrey’s equation requires that the deposited aerosol layer is uniformly distributed, thin and rigid (no liquids) where the former ensures that the symmetry of the loaded quartz crystal is identical to the pristine quartz (no shift in eigenfrequency due to change in symmetry) and the latter two characteristics requires that the entire deposited aerosol mass is perfectly coupled to the quartz. Often, a sufficiently thin layer refers to a deposited mass inducing less than 2% change in the eigenfrequency of the quartz (5 MHz quartz as used here), which translates to less than 0.1 MHz frequency change that corresponds to a maximum mass loading of 1770 μg/cm^2^ [54]. However, it could be lower for less tightly connected particle structures such as for instance “wobbly” agglomerates formed by entangles high aspect ratio materials as will be discussed in more detail in the discussion section.

**Fig. 1:**
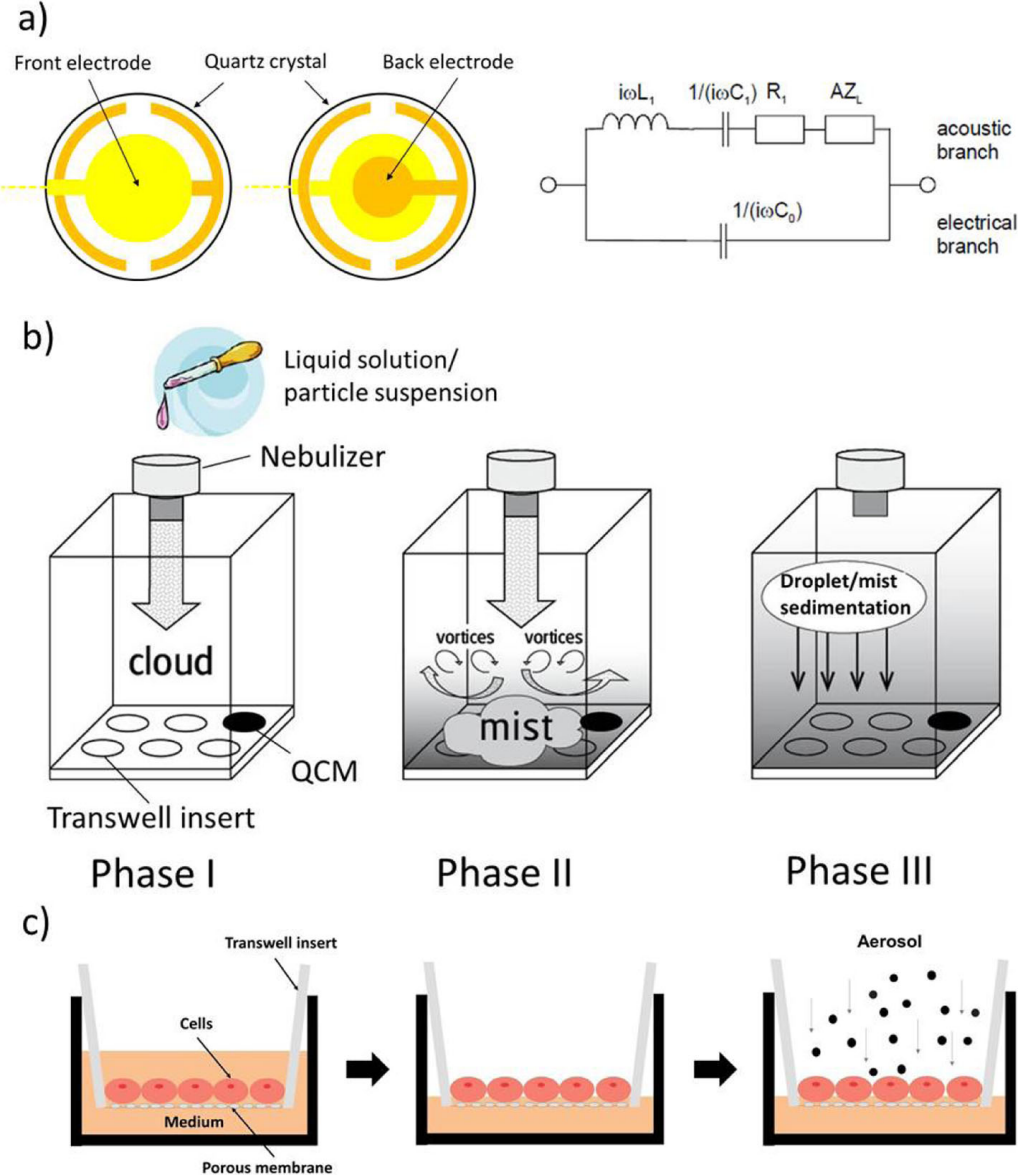
**a** Configuration of a typical crystal microbalance (QCM) and Butterworth-van-Dyke (BvD) equivalent circuit [56] (C_0_: electrical capacitance; C_1_: motional capacitance; L_1_: motional inductance; R_1_: motional resistance; A: effective area of the crystal; Z_L_: load impedance); **b** Nebulization and sedimentation of liquid solutions or nanoparticle suspensions using the VITROCELL**®** Cloud 6 system equipped with a quartz crystal microbalance (QCM) (adapted from Lenz et al. 2014 [29]); **c** Schematic view of cell cultivation on transwell inserts. First, cells are grown under submerged conditions (4d, until confluency) followed by air-lifting with a 24 h acclimatization phase and subsequent ZnO-aerosol exposure at the air-liquid interface

Based on Sauerbrey’s equation, the deposited mass can be calculated directly from the change in eigenfrequency and only well-known material constants. Thus, the QCM is a primary method for mass measurement (no calibration required) and it works independent of gravitational conditions, i.e. it can also be employed upside down or even in zero-gravity environments (e.g. space), since it detects inertial (not gravitational) mass. However, it is important to note that at this time QCMs cannot be employed for particle dosimetry under submerged cell culture conditions due to interference of viscoelastic effects from the cell culture medium. In this case the QCM measures the degree of viscoelastic coupling of the medium to the quartz crystal (i.e. viscosity) rather than deposited particle mass. A schematic diagram of the QCM electrical circuit is presented in Fig. 1a. The successful use of a QCM for particle dosimetry in a VITROCELL® Cloud type exposure systems (ALICE system) was firstly shown in 2009 [30, 57]. Photos of the current QCMs integrated in the VITROCELL® Cloud exposure systems are presented in Figure S1.

### VITROCELL® Cloud air-liquid interface cell exposure systems

The experiments in this study were conducted using the commercially available air-liquid interface cell exposure systems VITROCELL® Cloud 6 and Cloud 12 (VITROCELL Systems, Waldkirch, Germany) designed for simultaneous exposure of up to six transwell inserts (6-well size or smaller) and nine transwell inserts (12-well size or smaller), henceforth referred to as Cloud 6 and Cloud 12, respectively. If the QCM is included, the Cloud 6 and Cloud 12 can only be loaded with up to 5 and 8 inserts, respectively. A schematic depiction of the Cloud 6 is presented in Fig. 1b (analogous setup for Cloud 12), except that the Cloud 12 consists of a main and a sham control chamber with nine and three wells, respectively. The temperature of the exposure chamber can be controlled by a heating unit, which allows creating realistic physiologic conditions for the cells (here 37 °C). The wells are filled with cell culture medium to provide nutrient support to the cells during exposure from the basal compartment of the transwell inserts.

The principle of operation of the Cloud system is schematically depicted in Fig. 1b and has been previously described in detail [29]. In brief, cells are grown on 6-well or 12-well transwell inserts (for Cloud 6 and 12, respectively), which are subsequently placed into the wells at the bottom of the Cloud exposure chamber. Smaller inserts (12-well and 24-well inserts in Cloud 6, 24-well inserts in Cloud 12) can be hosted by the respective system using adapters. For aerosol-cell exposure, the substance to be tested is dissolved/suspended in an aqueous solution and 200 μl is nebulized with an Aeroneb Pro vibrating mesh nebulizer (Aerogen Inc., Galway, Ireland) located at the top of the chamber. The initially well-defined cloud of aerosol is ejected towards the bottom plate (Fig. 1b, *phase I*), diverted sideways and upwards at the bottom and lateral walls of the exposure chamber, respectively (*phase II*), and thus convectively mixed into a uniform mist which gravimetrically settles spatially uniformly onto the cells (*phase III*). The total exposure time is less than 5 min and the quartz crystal microbalance (QCM) is located in the corner well, but can be also placed in any well.

### Nebulization

The VITROCELL**®** Cloud systems are equipped with Aeroneb vibrating mesh nebulizers (Aerogen, Ireland), in which an aqueous solution or particle suspension passes through a vibrating mesh consisting of more than 1000 pores (ca. 10 μm in diameter), resulting in formation of dense cloud of micron sized droplets with mass median diameters of 2.5–6.0 μm. The metallic mesh, which vibrates at 128 kHz during nebulization, generates a typical aerosol output rate between 0.3 and 0.8 ml/min (saline).

Unless stated otherwise, the Cloud systems were operated according to manufacturer recommendation. This includes heating the Cloud system to 37 °C, filling the prescribed amount of medium into the wells of the Cloud systems, filling 200 μl of liquid into the nebulizer and allow for complete nebulization of this liquid (typically within less than 45 s). This is essential for obtaining reproducible results. It has been previously shown that efficiency and uniformity of aerosol deposition do not depend on the droplet size distribution generated by the nebulizer, since they are governed by cloud - not single particle - dynamics [29]. In addition, the nebulization and droplet formation processes did not significantly modify the particle size distribution in the liquid suspension (Figure S3 right).

### Determination of deposited dose and QCM accuracy

For determination of the accuracy of the QCM the measured QCM dose was compared to a reference value using spectrofluorimetric analysis of a water-soluble fluorescent tracer (fluorescein sodium salt; catalog no. 28,803; Sigma-Aldrich, St. Louis, MO, USA). For extremely low dose levels (below the detection limit of the fluorescein method), the proven repeatability of dose delivery with the Cloud systems was utilized for obtaining a reference dose as will be described below.

First, the wells of the Cloud system were filled with medium (14.5 ml for the Cloud 6 and 3.1 ml for the Cloud 12) and inserts were placed in all of the wells. This is important, since the performance of the Cloud system is different, if not all of the wells are occupied with inserts. For dosimetry measurements as performed here, either metal inserts from VITROCELL Systems were used or the medium in the wells was reduced by 0.5 ml both for the Cloud 6 and Cloud 12, respectively, to avoid direct contact of the membrane of the transwell insert with the medium (this would allow the fluorescein tracer to diffuse out of the insert into the basal medium, which biases the dosimetry measurement). Then the exposure chamber was closed, the QCM was started and due time was allowed for the QCM signal to stabilize (after the temperature of the quartz is stable near 37 °C). Subsequently, the QCM signal was set to zero, and another 0.5 to 1 min was allowed for obtaining a reliable zero-point measurement. Then, 200 μl of a saline solution (here either various concentrations of NaCl or Dulbecco phosphate buffered saline (DPBS, 10.56 mg/ml)) is nebulized in the Cloud system and the QCM-deposited aerosol volume is calculated from the QCM-detected mass of salt and the known salt concentration in the nebulized saline after the water was dried off (explained in more detail below). By spiking the saline solution with fluorescein sodium salt (15 μg/ml), the QCM-deposited salt mass can be determined by washing the QCM-deposited fluorescein-salt mixture from the quartz and subsequent quantitative fluorescence spectroscopy using a standard curve as described in detail below. After each measurement the surface of the quartz was gently and thoroughly wiped by a water or alcohol-wetted tissue and then dried with lab-grade tissue paper, to ensure that the deposited substance is fully removed. Additionally, before starting the next nebulization the users should wait for some time for the Cloud system temperature to return to the set body temperature range (typically only a few minutes).

From the deposited fluorescein dose the deposition factor (DF) of the Cloud systems was determined as described below, which indicates the fraction of the total nebulized fluorescein mass (contained in 200 μl of fluorescein solution) that reaches the bottom of the exposure chamber, i.e. DF is a measure of the combined losses in the nebulizer and to the walls (for no losses: DF = 1). The deposition factor is measured based on the (fluorescein) dose retrieved from the QCM (exposed area of the quartz crystal: 3.8 cm^2^) and the transwell inserts (4.5 cm^2^ for 6-well) by scaling the measured doses up to the entire surface area of the bottom of the chamber (Cloud 6: 144.5 cm^2^; Cloud 12: 136.5 cm^2^) (see eqs. 2 and 3). This implicitly assumes spatially uniform aerosol deposition, which has been demonstrated by Lenz and colleagues [29].

Customized Stainless Steel inserts (4.5 cm^2^, VITROCELL Systems, Wladkirch, Germany) without perforation of the bottom and similar geometries as the standard transwell inserts were used to ensure that the entire deposited aerosol remains in the insert for dosimetry analysis. Cells were not cultivated on this type of insert. For the Cloud 12, 12-well transwell inserts with pore size 0.4 μm were used, which did not leak liquid from the apical into the basal compartment. Fluorescein sodium salt dissolved in DPBS at different concentrations (diluted up to 1:50 with distilled water) were prepared and 200 μl were nebulized. Prior to nebulization, the (metal) inserts were pre-filled with a known volume of DPBS solution (Cloud 6/12: 0.6 ml/0.3 ml) for collection of the deposited fluorescein droplets. Subsequently, the fluorescein concentration in the DPBS solution was analyzed by fluorescence spectroscopy (Tecan Safire I, excitation, 483 nm; emission, 525 nm; Tecan Inc., Männedorf, Switzerland) and converted into DPBS dose using a fluorescein-DPBS standard curve. The amount of deposited fluorescein on the QCM was determined in the same way, from the wash-off of the crystal surface using a known amount of DPBS (Cloud 6/12: 0.6 ml/0.3 ml).

From the measured fluorescein concentrations in the liquids retrieved from the transwell inserts and the QCM crystal the corresponding deposition factor can be determined according to

2 $$ { Depositio nFactor}_{insert}=\frac{Depositio{n}_{measured}}{Depositio{n}_{max}}=\frac{C_{fluo. insert}\ast {V}_{sol. insert}}{C_{fluo. neb.}\ast {V}_{neb.}\ast \frac{A_{insert}}{A_{ch}}} $$

3 $$ { Depositio nFactor}_{QCM}=\frac{Depositio{n}_{measured}}{Depositio{n}_{max}}=\frac{C_{fluo. QCM}\ast {V}_{QCM wash- off}}{C_{fluo. neb.}\ast {V}_{neb.}\ast \frac{A_{QCM}}{A_{ch}}} $$

*C*_*fluo. Insert*_ *– fluorescein concentration in liquid retrieved from inserts determined by fluorescein spectrofluorimetry*

*V*_*sol. Insert*_ *– volume of DPBS solution pre-filled into the insert*

*C*_*fluo. neb.*_ *– known fluorescein concentration in nebulized suspension*

*V*_*neb.*_*– nebulized liquid volume*

*A*_*insert*_ *– insert area available for aerosol deposition (and cell seeding)*

*A*_*QCM*_ *– QCM crystal surface area available for aerosol deposition*

*A*_*ch*_ *– total bottom surface area of the exposure chamber*

*Deposition*_*measured*_ *– measured deposited solute mass by fluorescein spectrofluorimetry*

*Deposition*_*max*_ *– maximum possible deposited solute mass assuming all of the solute mass pipetted in the nebulizer is deposited evenly onto the bottom of the chamber (without any losses)*

The exact values of these parameters are provided in Table S1.

### Determination of QCM precision and limit of detection

**Fig. 2:**
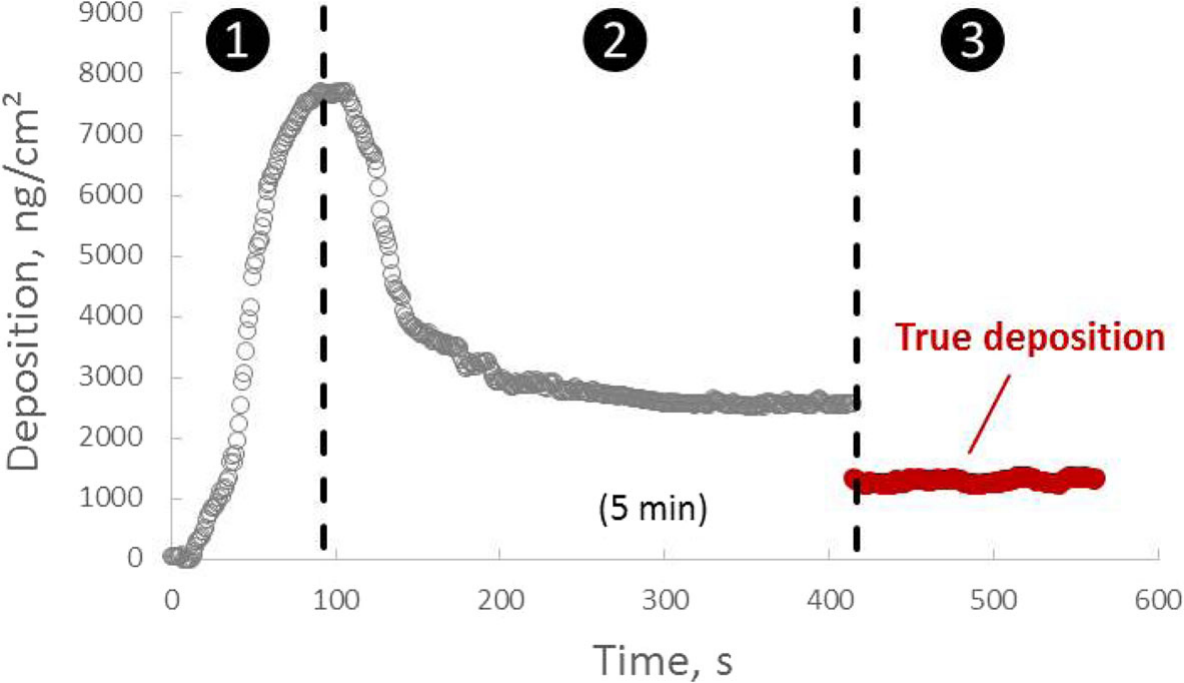
Typical QCM signal (1 Hz) observed during aerosol-cell exposure with the VITROCELL® Cloud system (here: 200 μl fluorescein solution was nebulized according to recommended operating conditions in the Cloud 6). The following three phases can be distinguished: *Phases I* and *II*: nebulization and aerosol deposition phase with closed chamber, respectively; *phase III*: Complete drying of the sample with chamber open (top part removed)

The precision of the QCM was defined as the variation (1σ) of the QCM signal under stable conditions (see *phase III* in Fig. 2). The QCM measures once every second (1 Hz), and 30 (Cloud 12) or 60 s (Cloud 6) of continuous measurements in the stable state were taken into account for calculating the variation. The different average times were selected to account for differences in electronic noise as explained below. Fluorescein nebulizations were repeated six times at different dose levels, and the average standard deviations for the two Cloud systems were taken as the mean variation (precision).

The lower limit of detection is typically defined as three times the 1σ noise level of a device. This can be determined by repeatedly measuring the variation of the device signals at the zero-point (or low-response level), which represents the noise level of the device. The zero-point stability has to be investigated for representative conditions observed during aerosol exposure. Since the Aeroneb nebulizers cannot nebulize pure deionized water (needs some free ions), we nebulized a very dilute salt solution (50-fold dilution of saline, i.e. 0.18 mg/ml; six nebulizations) according to the Cloud operating procedure described above and measured the signal variability (1σ) in the last 60 s of the measurement after the water has been dried off from the QCM (*phase III* in Fig. 2). The detection limit is defined as the 3σ level under these “near zero-dose” conditions.

### Comparison of dose delivered to QCM and transwell inserts

For assessment of the representativeness of the QCM dose measurement for the dose delivered to the transwell inserts (cells) the agreement of the doses delivered to both sites needs to be established. Fluorescein sodium salt was nebulized into stainless steel inserts (having very similar geometry as regular transwell inserts) placed in the wells of the Cloud systems. Again, the metal inserts were pre-filled with 0.8 ml and 0.3 ml for collection of the fluorescein aerosol in the Cloud 6 and Cloud 12, respectively. This allowed collection and spectrophotometric quantitative analysis of aerosol deposited in transwell inserts and on the QCM (SI Figure S1).

### Nanoparticle suspension preparation

As an application of the QCM, the dose-dependent toxicity of engineered zinc oxide (ZnO) nanoparticles (uncoated NM110 from the European Commission’s Joint Research Centre - JRC) was investigated under air-liquid interface exposure conditions. According to JRC, NM110 has a primary particle diameter of 158 nm and a BET (Brunauer–Emmett–Teller) specific surface area of 12 m^2^/g. A TEM (Transmission Electron Microscopy) picture showing the morphology of the particles is presented in Figure S2 (note that these are nanoparticles that suspended in liquid suspension before nebulization). This characterization by TEM shows the morphology of individual particles and also served as an additional quality control of the suspension to the DLS measurement.

The particles in powder form (6 mg and 12 mg for 2 mg/ml and 4 mg/ml, respectively) were suspended in 3 ml deionized water (Gibco, CAS-No. 15230162) in a flat-bottom glass beaker and pre-mixed with a vortex shaker. Subsequently, a probe sonicator (Bachofer GmbH, Reutlingen, Germany) was used to disperse the particles at an elevated energy level. An optimized sonication procedure was developed for these ZnO particles closely resembling the method described by DeLoid et al. [24]. The suspension was sonicated at 30% amplitude (corresponding to 3.7 W delivered power) for 160 s within in an ice bath to avoid heating of the suspension during sonication. Longer sonication durations will not decrease agglomerate size any further, but rather induce re-agglomeration of the particles (increased size). Immediately after probe-sonication, the particle size distribution of the suspension was measured by dynamic light scattering (Zetasizer Nano ZS Plus, Model Nr. ZEN3600, Malvern Instruments, U.K.). The ZnO size distribution in water is unimodal with a particle (agglomerate) volume/mass-median diameter of 290 nm and a geometric standard deviation of 1.44 (Figure S3). The suspension was stable for at least 4 h.

### Determination of target/delivered dose for ZnO toxicity study

The expected relevant dose range was assessed in view of the previously tested ranges from literature where significant cell responses were seen (e.g., IC50) [30, 34–40]. Moreover, the dose range should cover the onset dose at which cells start to show responses. This was determined as 0.1 to 1.5 cm^2^/cm^2^ (surface area of nanoparticles per surface area of cells exposed) or 0.83 to 12.5 μg/cm^2^ (mass of nanoparticles per surface area of cells exposed). Then the required amounts of ZnO suspension to be nebulized for obtaining five target doses in this range were selected based on eq. (5).

4 $$ {Dose}_{del. mass}=\frac{DF}{A_{ch}}\ast {Dose}_{neb}=\frac{DF}{A_{ch}}\ast {V}_{neb}\ast {C}_{sus} $$

5 $$ {Dose}_{del. surf}={Dose}_{del. mass}\ast {BET}_{part}\ast 10 $$

*Dose*_*del.mass*_ *– calculated delivered dose in mass, mg/cm*^*2*^

*Dose*_*neb*_ *– nebulized dose in mass, mg/cm*^*2*^

*Dose*_*del.surf*_ *–calculated delivered dose in surface area, cm*^*2*^*/cm*^*2*^

*A*_*ch*_ *– surface area of bottom of Cloud chamber, cm*^*2*^ *(Cloud 6: 145 cm*^*2*^*, Cloud 12 = 137 cm*^*2*^*)*

*DF – experimentally determined deposition factor (here 0.50; fraction of nebulized liquid depositing on chamber bottom).*

*C*_*sus*_ *– Concentration of nanoparticle suspension, mg/ml*

*V*_*neb*_*– nebulized volume of nanoparticle suspension, ml (here 0.2),*

*BET*_*part*_ *– mass-specific BET surface area of ZnO nanoparticles, m*^*2*^*/g*

The deposition factor (DF) represents the fraction of the nebulized liquid which deposits uniformly onto the bottom plate of the Cloud chamber. This value (here DF = 0.50) was determined by nebulization of fluorescein salt and quantitative fluorescence spectrometry as described by Lenz et al., 2014 [29]. In general, this value has to be determined experimentally for each nebulizer and each nebulized substance by solving eq. 2 for DF and measuring the delivered dose in the transwell inserts for any substance. According to our experience DF ranges between 0.5 and 1 depending on the output rate of the nebulizer.

Using eqs. 4 and 5 five doses spanning the target dose range and the associated Cloud operating parameters are presented in Table S2, where we worked with 2 mg/ml and 4 mg/ml suspensions and we took advantage of the fact that, albeit nebulization of 200 μl of liquid is recommended by us (and the manufacturer), the performance characteristics of the Cloud systems is essentially independent of the nebulized liquid volume between 75 and 1100 μl. These nebulized liquid volumes resulted in 1.05–15.75 μl deposited volumes per 6-well transwell insert (derived from 50% deposition factor for Cloud 6 system) which corresponds to a range of 2.6–37.5 μm liquid thin film thickness on the cell surface. This scenario is considered to be realistic for air-liquid interface exposure settings.

It is important to note that the deposition factor for a salt solution can differ from that of a nanoparticle suspension mainly due to potential partial retention of nanoparticles in the nebulizer. However, the nanoparticle-specific deposition factor for any given nanoparticle suspension (*DF*_*su*s_) can be readily determined using a QCM according to

6 $$ {DF}_{sus}=\frac{{\mathrm{Dose}}_{\mathrm{del}.\mathrm{mass}.\mathrm{QCM}}}{{\mathrm{V}}_{\mathrm{neb}}\ast {\mathrm{C}}_{\mathrm{sus}}/{\mathrm{A}}_{\mathrm{ch}}} $$

where the QCM-determined delivered mass dose (*Dose*_*del.mass.QCM*_ in mg/ml) is normalized to the nominally nebulized mass dose (denominator of eq. 6).

### Air-liquid-interface cell exposure

A schematic representation of the cell seeding and exposure conditions is shown in Fig. 1c. Human alveolar epithelial reported cells lie (A549), which was transfected with a luciferase reporter gene controlled by the IL-8 promoter [58], were cultivated on Corning® 6-well Transwell® inserts (Corning Inc., NY, USA) for 4 days (seeding density: 1 million/4.2 cm^2^) under submerged cell culture conditions to reach a confluent monolayer of cells. The cell culture medium used was Dulbecco’s Modified Eagle’s Medium (DMEM) supplemented with 10% fetal bovine serum (FBS), 2% penicillin and 2% gentamicin. The medium volume in the apical and basal compartment of the insert was 2 ml and 3 ml, respectively. After 4 days a confluent cell monolayer was reached and cells were air-lifted by removing the apical medium and reducing the basal medium volume to 1.2 ml which allows for medium-cell contact without exerting hydrodynamic pressure onto the cell layer. After an acclimatization period of 24 h at the air-liquid interface A549 cells were shown to form tight junctions and secrete a thin layer of surfactant on the apical side [59]. Subsequently, the cells were exposed to nanoparticle-containing aerosol droplets produced by nebulizing 100–1100 μl liquid suspension (Aeroneb Pro, output rate: 1 ml/min), in order to achieve the targeted dose range (see Table S2). Sham control experiments were performed with the same deionized water that was used for nanoparticle dispersion (spiked with 0.09 mg/ml NaCl to provide sufficient ions for proper operation of the vibrating mesh nebulizer). After exposure, the inserts were incubated for 24 h and then analyzed for viability (WST-1) and cytotoxicity (LDH, Lactate dehydrogenase release) as toxicological endpoints (see below). Three independent biological experiments were performed.

### Viability assay (WST-1)

Cell viability was determined from the cell proliferation reagent WST-1 (Roche Applied Sciences, Germany). The ready-to-use WST-1 reagent was 15-fold diluted with cell culture medium and added to the apical compartment of inserts. After 15 min of incubation at 37 °C the diluted WST-1 reagent was removed and light absorbance at 450 nm was determined with a standard plate reader (Tecan infinite M200 PRO, Grödig, Austria). The absorbance measurement was performed in triplicates. The IC50 dose is determined from the fitted dose response curve.

### Cytotoxicity assay (LDH)

The LDH cytotoxicity detection kit (Roche Applied Science, Mannheim, Germany) was used to measure the release of the intracellular enzyme lactate dehydrogenase (LDH), which is an indicator for cell membrane perforation. The test was performed on basal medium in triplicates. In each of the four wells in 96-well plate 30 μl basal medium was added into 70 μl fresh DMEM cell medium, and then 100 μl dye solution was added to all the wells. Subsequently the plate was wrapped up by aluminum foil and gently shaken for 15 min at 20 RPM (mechanical laboratory shaker, Heidolph Duomax 1030). At the end 50 μl HCL was added into the wells to stop the reaction. Afterwards the mixture was measured by a microplate reader at 492 nm wavelength (Tecan infinite M200 PRO, Tecan Austria GmbH, Grödig). The concentration of released LDH was then derived from a standard curve that covers a suitable concentration range. The maximum possible LDH level (positive control) was obtained from lysing the negative control cells (unexposed) with Glo-Lysis buffer (catalog no. E6120; Promega, Mannheim, Germany) and subsequent detection of LDH release. The results were normalized to the positive control and the negative control (LDH in basal compartment of unexposed incubator control) was subtracted to obtain a dose-response curve between 0 and 100% allowing for determination of the IC50 dose.

## Results

### Aerosol deposition on the quartz crystal microbalance (QCM) with the cloud system

Figure 2 depicts a typical QCM signal observed during nebulization of 200 μl (of fluorescein solution) and subsequent aerosol deposition in the Cloud 6 system. Three different phases can be distinguished:

- *Phase I*) At time 0 s the nebulizer is started, the aerosol cloud forms, gets mixed into a uniform mist rapidly filling the chamber from bottom to top and subsequent gradual setting onto the QCM with increasing rate reaching a maximum constant deposition rate (max. Slope of QCM signal) at about 30 s.
- *Phase II*) After reaching the peak of the QCM signal at about 90 s, the evaporation rate of deposited aerosol droplets exceeds the deposition rate of the cloud, leading to gradual decrease of the QCM signal until a stable value is reached at about 5 min. At this point the liquid film on the QCM has reached steady state conditions, i.e. no aerosol is depositing anymore and the air in the chamber is completely saturated, which prevents further evaporation of the deposited aerosol. The asymptotic value of the QCM at the end of *phase II* does NOT correspond to a correct mass. It rather correlates with the viscosity of the remaining liquid film due to viscoelastic effects of the film on the QCM performance. Only after complete drying, the QCM signal provides correct mass values.
- *Phase III*) The top of the cloud chamber is now removed, which allows the liquid layer on the QCM to dry out completely. The transition from liquid to solid phase typically occurs within a few seconds and causes a sudden drop or rise in the QCM signal depending on the applied solute dose. After this sudden drop/rise the signal remains on a stable level, which represents the true solute mass deposited onto the QCM. It is important to note that only in this phase (asymptotic value of *phase III*) the conversion from resonance frequency to deposited mass according to the Sauerbrey equation (eq. 1) is valid and the QCM reading represents the true deposited mass. The fact, that the QCM signal only represents true mass values after drying, prevents real-time measurement of aerosol deposition in the Cloud system with the QCM. Therefore, the QCM integrated in a Cloud system only allows for quasi-real-time dose measurements (dose measurement directly after the exposure when the sample has been dried).

It is important to note that complete drying of the deposited aerosol is a prerequisite for the QCM signal to provide a correct mass measurement, since residual liquid induces viscoelastic effects which result in an at least partial decoupling of deposited aerosol from the quartz crystal. The peak value of the QCM signal typically correlates well with the viscosity of the nebulized liquid (here water with less than 10 mg/ml salt) and not with the deposited mass of water or solute [30]. Similarly, the initial rise of the QCM signal during *phase I* can only serve as indication of aerosol deposition onto the QCM, not as real-time measurement of the deposited aerosol mass. All QCM data presented below were obtained after complete drying of the aerosol (*phase III* conditions).

### QCM accuracy

**Fig. 3:**
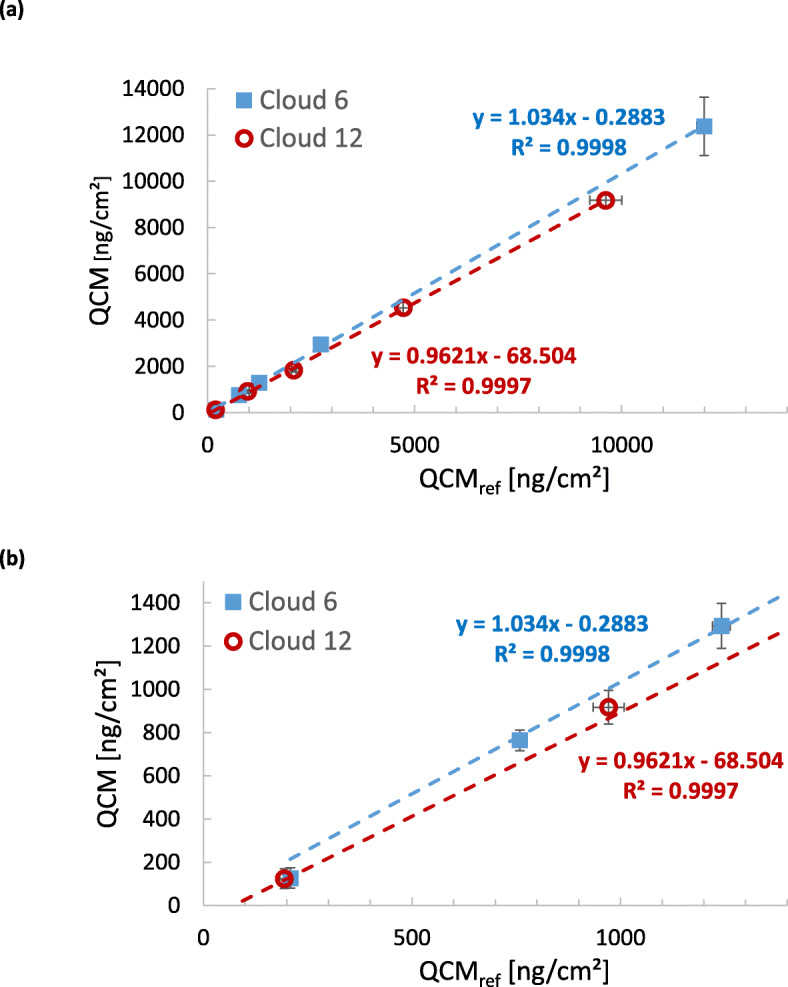
Measurement accuracy of the QCMs of the Cloud 6 and 12 system. The reference signal (QCM_ref_) is obtained from fluorimetric analysis of the deposited fluorescein salt. Panel **a** depicts the entire investigated mass range (ca. 100–15,000 ng/cm^2^) and panel **b** zooms in on the lower end of it highlighting the high degree of linearity of the QCMs over the entire dose range. (*n* = 6; Error bars represent 95% confidence level (2 SEM); note: error bars smaller than the symbol of the data point are not visible)

The accuracy of the QCM measurement was determined by comparing the QCM device signal with deposited salt mass obtained from fluorimetric analysis of QCM-deposited fluorescein-spiked salt. As shown in Fig. 3, there was excellent correlation (*R*^2^ = 1.000) between the two measurement methods over the tested dose range of from about 100 to 15,000 ng/cm^2^. F-test was used to determine whether the QCM results are able to predict the fluorescein deposition (SI, Table S3). This revealed that the accuracy of the QCM is 3.4% ± 0.9% and 3.8% ± 1.0% for Cloud 6 and 12, respectively.

### QCM precision and detection limit

**Fig. 4:**
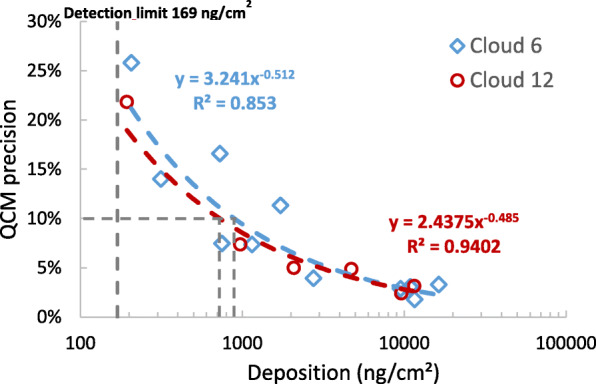
The relative precision (measurement uncertainty) of the QCM increases (decreases) with increasing delivered dose from ca. 25% at the lowest observed dose of 200 ng/cm^2^ (close to detection limit of 170 ng/cm^2^; see Table 1) to better than 10%, if the deposited dose remains above ca. 1000 ng/cm^2^ (> 892 ng/cm^2^ and > 724 ng/cm^2^ for Cloud 6 and Cloud 12, respectively)

The detection limit of an instrument depends on instrument “noise”, which can be experimentally determined from repeated measurements of the zero value. The detection limit is then defined as three times the noise level, i.e. three times the standard deviation about the zero value. Perfect zero values cannot be obtained with the Cloud system, since one cannot nebulize absolutely pure water with a vibrating mesh nebulizer, which needs a small amount of ions for stable operation. The lowest salt concentration without reduction in nebulizer output was found to be 0.20 mg/ml (1:50 diluted PBS), which resulted in a QCM-deposited mass dose of ca. 200 ng/cm^2^ (Fig. 4). The standard deviation of six consecutive exposures at the lowest possible salt dose was 56.5 and 56.2 ng/cm^2^ for the Cloud 6 and Cloud 12, respectively, implying a detection limit of 170 and 169 ng/cm^2^, respectively (Table 1; henceforth often approximated as 170 ng/cm^2^).

**Table 1:** The QCM detection limit is (close to) 170 ng/cm^2^ for Cloud 6 and in Cloud 12 using 60 s and 30 s of signal averaging time, respectively

	Cloud 6	Cloud 12
Detection limit, ng/cm^2^	170	169

For the QCM integrated in the Cloud system, instrument noise is mainly due to electronic noise of the QCM and instability of the QCM due to aerosol deposition with the Cloud system, which may induce small temperature changes in the quartz of the QCM (QCM is slightly temperature sensitive) associated with droplet deposition and subsequent evaporative cooling. The electronic noise level of the QCM (absolute standard deviation of QCM signal at zero level) can be derived from the variation of the 1 Hz signal values after drying (Fig. 2, *phase III*). As expected these values are relatively independent of the actually deposited dose as seen from Table S4 and Table S5**,** but here we report the near-zero values of 138.5 ng/cm^2^ and 10.7 ng/cm^2^ for the QCM in Cloud 6 and 12, respectively, since instrument noise is most relevant in the low dose regime. In order to assure that electronic noise contributes less than 10% to the observed total noise level of 170 ng/cm^2^, the QCM signal of the Cloud 6 was averaged over for 60 s (17.8 ng/cm^2^ = 138.5 ng/cm^2^/60^1/2^), while 30 s was deemed sufficient for the much less “noisy” QCM in the Cloud 12 system, which is mainly required to account for small drifts in the signal during *phase III* of the QCM measurement (Fig. 2). With these settings both QCMs have virtually identical detection limits (Table 1).

The measurement precision of the QCM was determined as the relative standard deviation of the QCM signal for doses ranging between ca. 200 ng/cm^2^ and 15,000 ng/cm^2^ (*n* = 6). The asymptotic value for large doses was determined as 2.3% (average precision for doses above 9500 ng/cm^2^), which is mainly due to the uncertainty in the reference measurement (quantitative spectrophotometric analysis of fluorescein). As for any instrument the precision decreases (higher variability) as the deposited dose decreases **(**Fig. 4**).** At a deposited dose of ca. 1000 ng/cm^2^ the precision of the Cloud 6 (892 ng/cm^2^) and Cloud 12 (724 ng/cm^2^) QCM reach 10%. If worse than 10% precision is acceptable, as is the case for many biological assays, the QCM can be applied to even lower dose levels down to the detection limit at 170 ng/cm^2^. For any given dose the expected QCM precision can be calculated from the fit equations given in Fig. 4.

### Comparison of QCM-delivered and cell-delivered dose

The prerequisite for using the QCM for dosimetry in cell culture experiments is that the QCM measures the dose delivered to the cells in the transwell inserts. Hence, agreement between QCM-reported and insert-delivered dose has to be verified.

**Fig. 5:**
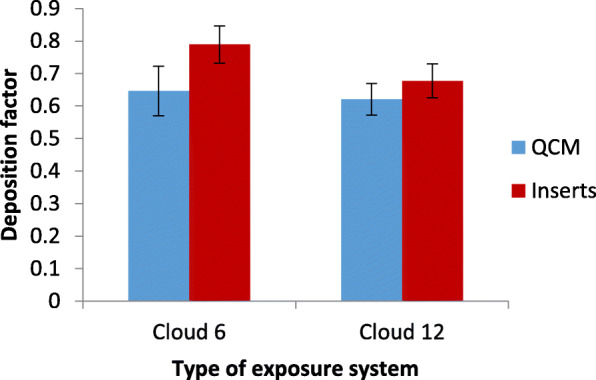
Deposition factors of QCM and transwell inserts showed no statistically significant difference for both Cloud systems (*p* = 0.06 and 0.08 for Cloud 6 and Cloud 12, respectively). Thus, the QCM determined mass dose is representative of insert−/cell-delivered dose. (*n* = 3; mean ± SD)

This was accomplished by determining the deposition factors on each of the inserts and on the QCM after nebulization of fluorescein-spiked saline, which allows for accurate dosimetry in the inserts (see eq. 2 and 3). As seen in Fig. 5 there is no statistically significant difference (*p* > 0.05) between insert-delivered and QCM-reported dose for both Cloud systems. It is important to note that this is only the case, if the Cloud systems are operated according to manufacture specifications, i.e. at 37 °C and with the prescribed volume of medium in the wells.

For the nebulizer used here (Aeroneb Pro; output rate of 0.4 ml/min) the mean (±SD) deposition factor was 0.79 (±5.8%) and 0.68(±5.2%) (average of all insert) for the Cloud 6 and 12, respectively, and there was no statistically significant dependence of the deposition factor on the position of the transwell insert (p > 0.05), i.e. all inserts received the same dose. The standard deviation of the deposition factor determined from the QCM signal of three independent experiments was 7.6 and 4.9% for Cloud 6 and 12, respectively. It is noteworthy, that in addition to the representativeness of the QCM dose for the average dose in the inserts (as shown in Fig. 5), but also low insert-to-insert dose variability is also an important aspect of the QCM providing accurate dose measurement for each of the inserts. This feature has already been investigated in two of our previous studies yielding an inert-to-insert dose variability (SD about the mean dose over all inserts) of 4.3% for the Cloud 6 [29] and between 3.1% and 8.3% for the Cloud 12 system (for different aqueous substances [60]). As these values are in agreement with the SD values shown in Fig. 5 it confirms that the QCM dose is representative for the dose delivered to each of the inserts.

### QCM-based dose-response curves for ZnO nanoparticles

**Fig. 6:**
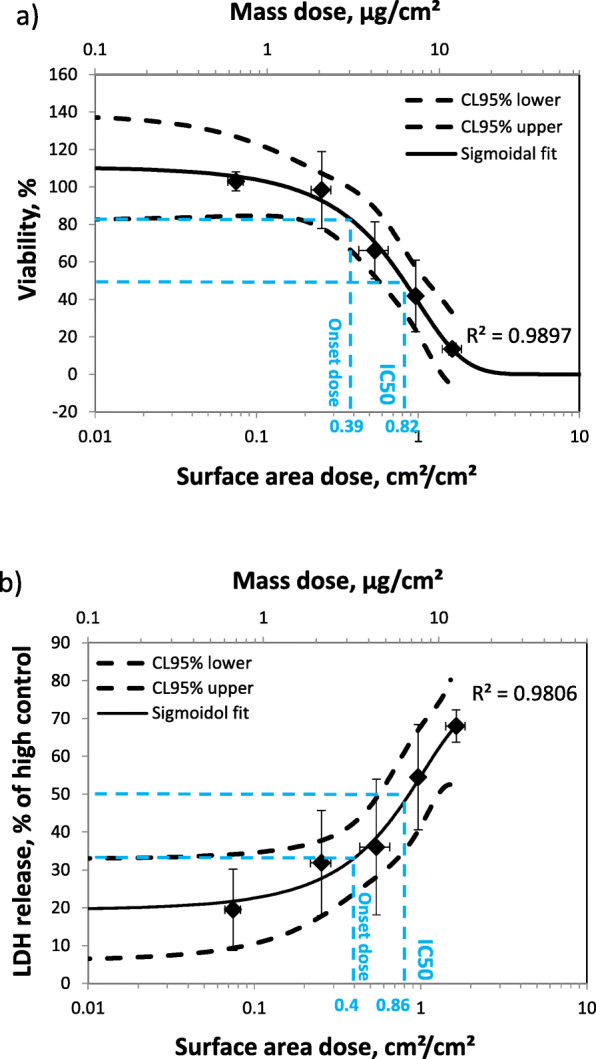
ZnO nanoparticle dose response curves for cell viability (WST-1) (**a**) and cytotoxicity (LDH release) (**b**). Conversion factor from surface area (cm^2^/cm^2^) to mass dose (μg/cm^2^) (secondary x-axis) is 8.3 (see eq. 7; biological replicates: *n* = 3)

As an application of the QCM, the Cloud 6 was used to expose lung epithelial cells (A549) to various doses of zinc oxide (ZnO) nanoparticles and the cell-delivered dose was measured with the QCM. Figure 6 shows the dose response curves 24 h after ZnO exposure for cell viability (WST-1) and cytotoxicity (LDH). The target doses and the actually delivered doses measured by the QCM using the operational parameters listed in Table S2 are presented in Table 2. The observed agreement between actual and target dose is within the previously reported dose repeatability of the Cloud system (< 20%; Lenz et al., 2014 [29]; Röhm et al., 2017 [60]). As expected, cell viability decreased (Fig. 6a) and cytotoxicity increased (Fig. 6b) for elevated doses. The dose-response patterns were fitted to sigmoidal curves (*R*^2^ > 0.98), which allows determining of both onset andIC50 doses. The onset doses were determined from the 95% confidence level curves (CL95). The onset and IC50 doses were about 0.40 and 0.84 cm^2^/cm^2^ (or 3.33 and 7.00 μg/cm^2^) independent of the toxicological endpoint (viability, cytotoxicity) (Table 3). Since cytotoxicity is considered a more severe insult than viability loss one might have expected that the characteristic doses for cytotoxicity are higher than those for reduced viability. However, this is not the case which might be due to the relatively steep dose-response curves and the fact that Zn ions are actively regulated by the cells. Once the metabolic buffering breaks down cells loose viability and experience cytotoxicity almost immediately, i.e. at very similar dose levels.

**Table 2:** Target dose range and actually delivered doses measured by QCM

Target dose, cm^2^/cm^2^ (μg/cm^2^)	0.1 (0.83)	0.3 (2.5)	0.5 (4.17)	0.9 (7.5)	1.5 (12.5)
Actual dose, cm^2^/cm^2^(μg/cm^2^)	0.080 (0.63)	0.25 (2.11)	0.54 (4.52)	0.97 (8.07)	1.64 (13.6)
Stand. Dev., cm^2^/cm^2^	0.01	0.04	0.11	0.05	0.22

**Table 3:** Onset and IC50 doses are almost independent of the toxicological endpoint (WST-1 and LDH)

Toxicol. endpoint	Onset dose, cm^2^/cm^2^ (μg/cm^2^)	IC50, cm^2^/cm^2^ (μg/cm^2^)
Viability (WST-1)	0.39 (3.25)	0.82 (6.83)
Cytotoxicity (LDH)	0.40 (3.33)	0.86 (7.17)

### Toxicological onset doses for nanoparticles from previous studies

**Fig. 7:**
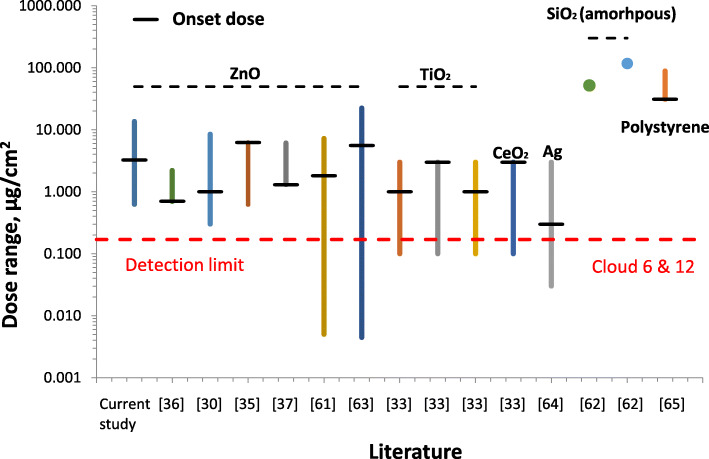
Reported dose ranges for particle-cell exposure experiments at the air-liquid interface from literature [30, 33, 35–37, 61–65] using different biological endpoints. The respective onset doses, which are indicated by horizontal black lines, are above the detection limit of the QCMs of the two Cloud systems (0.17 μg/cm^2^; red dashed line)

For a more comprehensive view on toxicologically relevant onset doses, the literature was reviewed for studies of air-liquid interface cell exposure with various types of nanomaterials, cell types and toxicological endpoints including viability, cytotoxicity and pro-inflammation markers (Fig. 7). The deposited aerosol doses were taken as reported by the authors of these studies relying on various on-line and off-line characterization techniques, such as QCM [33, 35], atomic absorption spectroscopy [30, 34–40]), SEM and TEM [61, 62], ICP-MS [33] as well as fluorescence analysis [62]. For the two types of amorphous SiO_2_ nanoparticles a significant release of LDH (Aerosil 200, green point) and pro-inflammation (IL-8 release) was observed (50 nm SiO_2_; light blue point) [62], but an onset dose could not be specified, since only one dose was tested. For other metal oxides such as ZnO, TiO_2_, CeO_2_ and Ag none of the studies reported onset doses below the detection limit of the Cloud QCMs (170 ng/cm^2^). Not even the analysis of the more sensitive mRNA induction of oxidative stress and proinflammatory markers resulted in onset doses below 0.7 μg/cm^2^ for ZnO nanoparticles [36]. From our analysis of literature data we conclude that the QCMs of the Cloud systems are sufficiently sensitive for toxicological onset doses observed for common types of nanoparticles (e.g. ZnO, TiO_2_, CeO_2_, Ag, SiO_2_ (amorphous), polystyrene), albeit the onset dose of Ag (0.3 μg/cm^2^) is just 1.5-fold about the detection limit of the QCM. Thus, it is conceivable that for certain materials with extremely high mass-specific toxicity the detection limit of the VITROCELL Cloud QCMs should be further improved (some possibilities are given in the discussion).

## Discussion

Delivery of high enough doses within a relatively short period of time and exact knowledge of the cell-delivered dose are critical for efficient and reliable in vitro toxicity screening of nanoparticles [66]. For optimized workflow and due to the limited lifetime of many cell culture models the target dose should be delivered within a few hours (or faster). Accurate knowledge of the biologically relevant cell-delivered dose (rather than the nominal applied/nebulized dose) is the prerequisite for reliability of the reported onset or IC50 doses and comparability with other studies. The VITROCELL® Cloud systems meet both of these requirements. The fact that the QCM dries off completely within seconds after opening the exposure chamber to ambient air indicates that the liquid phase of the aerosol layer deposited onto the cells will also evaporate prior to putting the inserts back into the incubator. This is an important aspect, since it suggests that the additional thin liquid layer deposited on the cells will not interfere with air-liquid cell culture conditions.

VITROCELL® Cloud systems allow for rapid and efficient delivery of high doses of nanoparticle to air-liquid interface cell cultures, which are high enough for determination of onset and IC50 doses of toxicological effects such as expression of oxidative stress and pro-inflammatory markers on the mRNA and protein level as well as cell viability (WST-1) and cytotoxicity (LDH). It was shown that specific target doses (here for ZnO nanoparticles) can be reached within a margin of 20% (95% CL) in a fast (typically < 5 min) and reproducible manner (SD of 7.6 and 4.9% for Cloud 6 and 12, respectively). This is consistent with the 18.6% dose repeatability (95% CL) observed by Lenz et al. 2014 [29] for a prototype version of the VITROCELL® Cloud 6 system. Also the deposition factor of 0.79 observed here (fractional dose reaching the bottom of the exposure chamber) is reasonably close to the value of 0.85 reported by Lenz et al. 2014 for a different Aeroneb nebulizer [29]. Similarly, Röhm et al. 2017 [60] reported deposition factors between 0.84 and 1.0 for a VITROCELL® Cloud 12 using salt and protein solutions for nebulization. According to our experience, the mean deposition factor of Cloud systems is ca. 0.85 (0.7 to 1.0) unless the nebulizer output rate reaches relatively high values near 0.8 ml/min, which may be associated with lower deposition factors as low as ca. 0.50 as seen here. Assuming a representative mean deposition factor of 0.85 one finds that 2.5 and 0.7% of the nebulized substance is deposited in each 6-well or 12-well transwell insert (BD Falcon) with a cell-covered area of 4.2 and 1.12 cm^2^ for the Cloud 6 and Cloud 12 system, respectively. Thus, having a full set of inserts in place (QCM is taken out) up to 15% (6 inserts) and 6.3% (9 inserts) of the nebulized substance can be deposited onto the cells with the Cloud 6 and 12, respectively. With the QCM in place these values are reduced to 12.5 and 5.6%, respectively.

QCMs measure the inertial (not gravitational) mass deposited onto a quartz crystal from first principles of physics by relating the change in resonance frequency of the quartz crystal with its change in mass according to the Sauerbrey equation (eq. 1). Unlike gravitational measurement methods QCMs work independent of gravity, i.e. they can also be operated upside down or in space. More importantly, − as any first-principle method - QCMs do not need to be calibrated, since the relationship between measured frequency shift and deposited mass is uniquely defined by the geometry and by well-known material constants of the quartz crystal, hence it does not require any calibration factors. However, the Sauerbrey equation only applies under certain conditions, which include spatially uniform deposition of the mass onto the quartz and perfect coupling of the deposited mass with the quartz (i.e. no viscoelastic effects as observed for liquids). The latter requires complete drying of the aerosol deposited onto the quartz (only *phase III* QCM data are reliable; Fig. 2) and the former was experimentally verified by obtaining agreement between QCM-measured and reference mass within statistical uncertainties. On the other hand, it has recently been show that QCMs allow precise measurements for non-uniformly distributed nanoparticle mass, if the mass is deposited symmetrically in a very narrow region around the center of the quartz crystal [67]. In any case, occasional verification of the accuracy of the QCM using the fluorescein-based method presented here is recommended as quality control to avoid inadvertent measurement errors due to e.g. a damaged quartz crystal or an electronic failure in the electronic system of the QCM. It is also important to note that QCMs are sensitive to temperature and external vibrations. Thus, temperature stability and vibrational isolation of the QCM is essential for measurement accuracy. In particular for aerosol-cell exposure systems with low particle deposition rate long exposure times may give rise to zero-point drifts, which result in reduced measurement accuracy.

In this study we present a method for determination of detection limit and accuracy of QCMs which are integrated in ALI cell exposure system for real-time dosimetry of aerosolized particles. The QCMs of the two VITROCELL® Cloud systems show essentially the same performance characteristics provided the higher electronic noise of the Cloud 6 is compensated for by averaging the QCM signal over 60 s as compared to 30 s for the QCM of the Cloud 12. During *phase III* some slow (40 s periodicity), low amplitude oscillation of the QCM signal was observed (Fig. 2), which is likely due to the heating-cooling cycle of the temperature control system integrated in the base block of the Cloud system for maintaining 37 °C. This can be eliminated by closing the chamber again after complete drying of the sample and giving it ca. 1 min for thermal equilibration. The observed optimum QCM accuracy and precision of better than 4 and 3% (for large doses), respectively, is very close to the experimental measurement uncertainties of the reference dosimetry method due to uncertainties in pipetting and fluorescence intensity measurement. Thus, for high doses the QCM is likely to be more precise than reported here. For low dose exposures, QCM measurements are associated with lower precision down to ca. 30% at the detection limit (170 ng/cm^2^), which is often acceptable, since many biological assays are also not more precise than 30%. However, if more accurate dosimetry is required, longer averaging of the QCM signals (up to 3 min seems reasonable) and careful determination of the zero-offset (average signal just prior to nebulization – possibly also extended to 3 min) are recommended. Also *n* repeat exposures will enhance the precision of mean QCM dose to *30%/sqrt(n)*.

For low toxicity materials, doses larger than the 12 μg/cm^2^ investigated here may be required to induce toxic effects. We have shown previously, that 5 MHz quartz crystals (as used here) should have a linear response range of up to 1770 μg/cm^2^, which was experimentally verified up to 160 μg/cm^2^ [30]. Considering that 1770 μg/cm^2^ corresponds to an at least 5 μm thick nanoparticle layer (assuming an effective (volume) density of 3.5 g/cm^3^, which is reasonable upper limit for agglomerated spherical metal oxide particles). This typically corresponds to several monolayers of nanoparticles, which is likely the biologically relevant upper mass dose, since higher doses will not enhance the dose getting in touch with the cells due to steric hindrance. For spherical nanoparticles, it was shown that 5 MHz QCM are linear up to at least 160 μg/cm^2^ [30]. However, for some nanomaterials the effective density is much lower, such as for entangled high aspect ratio nanomaterials (especially fibers). In this case, the deposited thicker layer may result in non-perfect coupling of the deposited nanomaterials with the quartz crystal resulting in a negative bias in the QCM-determined mass measurement at much lower values than 1770 μg/cm^2^. However again, steric hindrance will likely limit the toxicologically effective mass dose to values much lower than the upper limit of the linear detection range of the QCM.

For highly toxic substances the lower detection limit of 170 ng/cm^2^ is the most critical parameter. Considering that an atomic monolayer (of e.g. NaCl) corresponds to a dose of 18 ng/cm^2^ a QCM sensitivity of 170 ng/cm^2^ allows detection of about 10 atomic layers (ca. 1 nm thin layer, density ~ 2 g/cm^3^). While we have shown that this is sufficient to determine the onset doses of even the nanoparticles with the highest toxicity presented in Fig. 7, namely ZnO and Ag nanoparticles with onset doses of 700 ng/cm^2^ and 400 ng/cm^2^, respectively, it is conceivable that lower onset doses may be required for some extremely highly toxic particles and/or some very sensitive cell-based biological response systems. For instance ZnO nanoparticles with a very similar BET surface area as used here (13 m^2^/g), responded at up to 5-fold lower doses of between 0.7 and 2.5 μg/cm^2^ to mRNA induction of oxidative stress and pro-inflammatory genes [30, 36] as compared to the 3.3 μg/cm^2^ reported for cell viability and cytotoxicity in this study(. Also enhanced mass-specific BET surface area could lower the onset dose to values below the QCM detection limit of 0.17 μg/cm^2^ [68–70].

However, if detection limits lower than 170 ng/cm^2^ should be required, the averaging time for QCM measurements can be prolonged and the more advanced offset correction technique as indicated above for improved QCM accuracy can be applied. Moreover, technical improvements of the experimental procedure may focus on reducing electronic noise, enhancing temperature stability and avoiding artifacts due to adsorption of gaseous compounds such as water vapor (reducing relative humidity) and non-perfect isolation of the quartz of the QCM from external vibrations. Last but not least, the VITROCELL® Cloud systems offer another alternative for accurate dose assessment even below the detection limit of the QCM. The linear relationship between concentration of the nebulized nanoparticle suspension and deposited mass dose (as evidenced by Fig. 3) can be extrapolated to infer the cell-delivered dose below the detection limit of the QCM. This can be pursued by deriving the suspension-specific deposition factor (*DF*_*sus*_*)* from the QCM measurement (see eq. 6) and then eq. 5 can be used to determine the cell-delivered dose from the known nanoparticle concentration and *DF*_*sus*_. We note that this approach is justified, since the Cloud systems work highly reproducible (ca. 9% dose variability from nebulization to nebulization; data not shown, but consistent with Lenz et al., 2014 [29]) and independent of the concentration of the nebulized suspension (can be seen from the linear QCM-to-reference mass dose curves in Fig. 3). We remind the reader, that the deposition factor for a salt solution can differ from that of a nanoparticle suspension mainly due to potential partial retention of nanoparticles in the nebulizer. Hence, *DF*_*sus*_ has to be used for dose extrapolation to below the detection limit of the QCM.

Considering the increasing body of evidence on surface area as most relevant dose metric for particle toxicity studies [13, 71] it is important to convert the mass-based detection limit of the QCM into equivalent surface area-based detection limit. For a given mass-specific BET surface area, this can be accomplished according to

7 $$ {Detection\ limit}_{QCM}(SA)={SA}_{BET}\left(\frac{m^2}{g}\right){Detection\ limit}_{QCM}/100, $$

*Detection limit*_*QCM*_(*SA*): surface area-based detection limit of the QCM (given in cm^2^/cm^2^)

*Detection limit*_*QCM*_: mass-based detection limit of QCM (here 0.17 μg/cm^2^)

*SA*_*BET*_: mass-specific BET surface area, m^2^/g

**Fig. 8:**
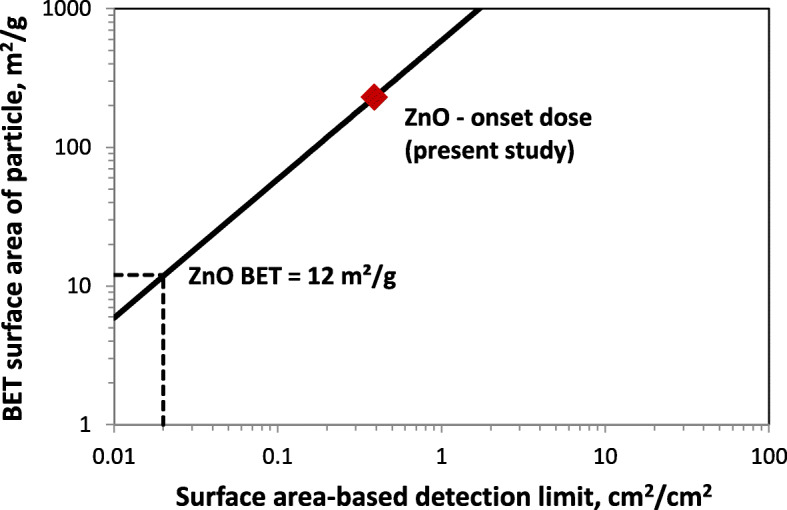
Conversion of mass-based QCM detection limit of the CLOUD systems (170 ng/cm^2^) into corresponding surface area-based QCM detection limits for mass-specific BET surface area values between 6 and 1000 m^2^/g (see eq. 7)

For the ZnO nanoparticles used here (*SA*_*BET*_ = 12 m^2^/g) and the mass-based detection limit of 0.17 μg/cm^2^ the corresponding surface area-based detection limit is 0.020 cm^2^/cm^2^, which is clearly below the detected onset dose of 0.39 cm^2^/cm^2^ (Table 3). On a more general level Fig. 8 depicts how the surface area-based detection limit varies with the mass-specific BET surface area for a (constant) mass-based detection limit of 170 ng/cm^2^ (as for Cloud 6 and Cloud 12). Obviously, larger mass-specific BET surface area shifts the surface-area based onset dose to larger values, i.e. the QCM becomes less sensitive in terms of surface-area based onset dose.

There are numerous analytical techniques for particle dosimetry employed in ALI exposure studies, which include ICP-MS, atomic absorption spectroscopy (AAS) and electron microscopy (e.g., SEM and TEM). All of these methods have advantages and limitations, which cannot be discussed in detail here. However, in comparison to these analytical techniques, QCMs feature some major advantages. Firstly, QCMs provide (quasi-) real-time dose characterization during the course of the cell exposure experiment without sophisticated instrument handling or sample extraction/preparation procedures. QCMs are relatively cost- efficient (a few thousand dollars) and suitable for all types of solid materials and solutes (even mixtures thereof), provided the solvent can be dried off reasonably quick (ideally within minutes or at least tens of minutes) to avoid viscoelastic decoupling of the deposited material from the quartz crystal. Even somewhat gel-like materials such as organic carbon compounds contained in cigarette smoke particles can be measured, if their viscosity is high enough to avoid viscoelastic decoupling [72]. Lastly, as first-principle method, QCMs do not require calibration, but instrument performance can be readily verified with for instance fluorescent particles as described here. On the other hand, QCMs cannot detect elemental composition of nanoparticles actually deposited on or taken up by cells, since nanoparticles need to deposit directly onto the quartz crystal. Also some other analytic techniques offer lower detection limits, if cross-sensitivity or interference with cell debris can be avoided (e.g., carbonaceous nanoparticles contained in cell pellets are difficult to detect with elemental detection techniques like ICP-MS or AAS. QCMs suffer from reduced detection limit for long-term cell exposure studies (longer than a few hours) due to temperature- and electronically-induced offset drifts. In summary, while QCMs have their limitations, they are a time- and cost-efficient tool for real-time measurement of the cell-delivered nanoparticle mass in air-liquid interface cell exposure experiments with sufficient sensitivity and accuracy for many of the currently investigated types of nanoparticles.

One additional precaution for correct operation of the Cloud systems is to ensure the quality of the nanoparticle suspension prior to nebulization. Although the pore diameter of the Aeroneb nebulizers employed by the Cloud system are generally large enough (4–10 μm) for nanoparticles (and their agglomerates) to pass through, for suspensions containing larger than ca. 3 μm particles or agglomerates the nebulizer pores may get blocked. If this occurs, the output rate of the nebulizer may get reduced to the point where the kinetic energy of the cloud is insufficient to provide sufficient convective mixing to ensure uniform aerosol deposition in the sedimentation chamber, which renders the QCM measurement non-representative for the nanoparticle dose delivered to the transwell inserts (cells). Hence, extra care should be given to materials that larger than 3 μm in at least two dimensions, are difficult to disperse and/or form entangled structures (e.g. some tube-like and fiber-like particles). Remarkably, well-dispersed non-entangled high-aspect ratio tube−/fiber-like particles up to 50 μm long have been successfully delivered to cells with Cloud-type ALI systems [31]. Moreover, clogging of the nebulizer pores may also occur for any type of particle, if the concentration of the particle suspension used for nebulization is too high (typically ca. 5 to 10 mg/ml). While this may lead to lower aerosol deposition factors which is reflected by reduced QCM values, this is not a fundamental problem as long as the nebulizer output rate is not dropping too low for uniform aerosol deposition in the exposure chamber. Even early onset of clogging can be detected by a reduced deposition factor as compared to that of a salt solution.

It is important to note that the results from this study on the VITROCELL Cloud QCMs provide guidance on how to assess the suitability of QCMs as dosimetry tool for other aerosol-cell exposure systems as well. As demonstrated here, the key parameters for this assessment are the lower limit of detection of the QCM, the onset dose of the toxicological assay and the aerosol dose deposited onto the QCM (cells) during the exposure. Repeated sham exposures under identical conditions as observed during aerosol exposure (here: nebulization of pure water with the lowest possible amount of salt to ensure stable operation of the nebulizer (0.01% w/v NaCl)) will reveal the mean and standard deviation (SD) of the zero-point (zero-point stability). The (lower) limit of detection (LoD) of the QCM can then be calculated from LoD = mean + 3*SD and the QCM is suitable for dose-response studies, if the onset dose of the used toxicological assay is above the LoD. While experimental determination of the LoD of the QCM is relatively simple (as described here), the onset dose (sensitivity) of the toxicological assay, which depends amongst others on cell culture model, toxicological endpoint and time point of investigation, can only be detected, if the cell-deposited aerosol dose is large enough to exceed the toxicological onset dose. This strongly depends on the aerosol-cell delivery rate which can be obtained with an aerosol-cell exposure system under the specific conditions during aerosol-cell exposure.

The literature review of air-liquid interface cell exposure studies presented in this study demonstrated that – at least for the LoD of the VITROCELL Cloud systems (170 ng/cm^2^) - even high toxicity/hazard materials such as ZnO and Ag nanoparticles can be assessed with QCMs. However, this may not be the case for nanoparticles with even higher mass-specific toxicity or for aerosol-cell exposure conditions, which induce large zero-point drifts in the QCMs signal resulting in increased LoD values (less sensitivity). While the former cannot be influenced by experimental design, the latter can be optimized by tightly controlling temperature and humidity conditions of the aerosol-laden air, minimizing mechanical vibrations transferred to the QCM (vibration dampening) and exposure time, which can be reduced by enhancing aerosol-cell deposition efficiency (fractional aerosol deposition), aerosol concentration and aerosol flow rate. On the other hand, mass-specific nanoparticle toxicity depends on numerous parameters including material type (chemistry, crystallinity, surface charge, surface functionalization), mass-specific BET surface area, particle size (affects cellular uptake and biokinetics) and shape (fiber-like is often more toxic than spherical shape). Understanding of the relevance of all of these parameters allows optimization of the experimental conditions for sensitive and accurate QCM-based real-time dosimetry during air-liquid interface aerosol-cell exposure experiments.

## Conclusions

This study provides evidence that QCMs are suitable for real-time dosimetry in particle toxicology studies with cell cultures under air-liquid interface conditions. An experimental method for determination of LoD (lower limit of detection), accuracy and precision of QCMs using a fluorescent tracer (fluorescein salt) was presented and applied to the QCMs integrated in the VITROCELL® Cloud 6 and Cloud 12 aerosol-cell exposure systems. The QCMs of the Cloud systems (LoD = 170 ng/cm^2^) are sensitive enough for hazard assessment of a wide variety of nanoparticles even for highly toxic NM110 ZnO and Ag nanoparticles. This is generally the case, if the LoD of the QCM (here 170 ng/cm^2^) is below the toxicological onset dose of the specific material under investigation, which depends on numerous parameters including material type, particle size, shape, mass-specific BET surface area as well as nanoparticle dose rate, toxicological endpoint, time point of investigation and cell type. Hence, the detection limit of a QCM should be determined for each ALI exposure system and QCM. Although QCMs do not need to be calibrated, occasional verification of the detection limit and accuracy of the QCM using the fluorescein-based method presented here should be performed as quality control measure to avoid inadvertent measurement errors due to e.g. a damaged quartz crystal. Comparing to other dose characterization techniques used in ALI exposure studies, QCMs make it possible to measure cell-delivered doses in (quasi-) real-time, and being at the same time material-independent and user friendly.

The Cloud 6 system was employed to perform dose-controlled exposure of alveolar lung epithelial cells (A549) to ZnO nanoparticle (NM110) under ALI conditions. Each exposure took about 10 min and an onset dose of 3.3 μg/cm^2^ (or 0.4 cm^2^/cm^2^) was found for both cell viability (WST-1) and cytotoxicity (LDH) as toxicological endpoint. The corresponding IC50 values were 6.83 μg/cm^2^ (or 0.82 cm^2^/cm^2^) and 7.17 μg/cm^2^ (or 0.86 cm^2^/cm^2^) for WST-1 and LDH, respectively. The onset dose of 3.3 μg/cm^2^ is in-between previously reported ALI onset doses for ZnO nanoparticles (0.7–7 μg/cm^2^). Unlike many submerged cell culture data, these values reflect actual cell-delivered doses. This 10-fold variability of onset doses is therefore not due to bias in cell-delivered dose, but possibly due to differences in mass-specific (BET) surface area, the choice of cell type and toxicological endpoints as well as the dose rate of ZnO delivery. Thus, future research could utilize the Cloud systems to investigate the relevance of these parameters for toxicological onset doses.

## Supplementary information

**Additional file 1: Table S1.** Parameters for calculation of deposition efficiency (Cloud 6). **Table S2.** Target doses and associated conditions of suspension and nebulization to be adopted during cell exposure. **Table S3.** Results of F-test on correlations between QCM measurements and fluorescein depositions for both Cloud systems. **Table S4.** Electronic noise induced precision (relative standard deviation (SD)) and lower detection limit (3 SD) of the VITROCELL® Cloud 6 for 1 Hz QCM data (for 60 s averaged QCM signal) for fluorescein-spiked and pure salt nebulizations. **Table S5.** Electronic noise induced precision (relative standard deviation (SD)) and lower detection limit (3 SD) of the VITROCELL® Cloud 12 for 1 Hz QCM data (for 30 s averaged QCM signal) for fluorescein-spiked and pure salt. **Figure S1.** – Photos of the QCM setups in the VITROCELL® Cloud 6 (upper-left) and 12 (upper-right) and corresponding 6-well and 12-well stainless steel inserts for fluorescein deposition (lower-left and lower-right), respectively. **Figure S2.** TEM pictures of ZnO NM110 (nanoparticle suspension prepared in distilled water before nebulization). **Figure S3.** Left: Particle volume-size distribution of ZnO nanoparticles dispersed in water revealed volume/mass median diameter and geometric standard deviation of 290 nm of 1.44, respectively (dynamic light scattering, suspension concentration: 1 mg/ml). Right: Comparison of the volume-size distributions before and after nebulization (0.5 mg/ml, liquid droplet was collected in an eppendorf tube upon nebulization and subsequently measured by DLS). Volume/mass- median diameter: 256.7 (before) and 272.7 (after) nm. **Figure S4.** QCM stability (1 Hz data) at zero-point level (unloaded QCM of Cloud 6) under thermal equilibrium (ca. 37 °C). Three repeated measurements were conducted (T1, T2, T3) for 1 h (3,600 data points each). If the zero point of the QCM is set by the operator just prior to the experiment at an “arbitrarily” selected data point, this can result in a “false” mean zero point level (here between − 53.4 and + 47.3 ng/cm^2^ (Table S6). **Table S6.** Numeric evaluation of zero point measurements depicted in Figure S4. **Figure S5.** Equivalent to Figure S4, but for the Cloud 12 system with a “false” mean zero point level between 3.5 and 21.8 ng/cm^2^ (Table S7). **Table S7.** Numeric evaluation of zero point measurements depicted in Figure S5.

## Data Availability

All data generated or analyzed during this study are included in this published article and its supplementary information files.
