# Supplementary material for: Quartz crystal microbalances (QCM) are suitable for real-time dosimetry in nanotoxicological studies using VITROCELL®Cloud cell exposure systems
Source: Part Fibre Toxicol. 2020 Sep 16;17:44. doi: 10.1186/s12989-020-00376-w (PMC7493184; doi:10.1186/s12989-020-00376-w)
Supplement: Supplementary file 1 — Additional file 1: Table S1. Parameters for calculation of deposition efficiency (Cloud 6). Table S2. Target doses and associated conditions of suspension and nebulization to be adopted during cell exposure. Table S3. Results of F-test on correlations between QCM measurements and fluorescein depositions for both Cloud systems. Table S4. Electronic noise induced precision (relative standard deviation (SD)) and lower detection limit (3 SD) of the VITROCELL® Cloud 6 for 1 Hz QCM data (for 60 s averaged QCM signal) for fluorescein-spiked and pure salt nebulizations. Table S5. Electronic noise induced precision (relative standard deviation (SD)) and lower detection limit (3 SD) of the VITROCELL® Cloud 12 for 1 Hz QCM data (for 30 s averaged QCM signal) for fluorescein-spiked and pure salt. Figure S1. – Photos of the QCM setups in the VITROCELL® Cloud 6 (upper-left) and 12 (upper-right) and corresponding 6-well and 12-well stainless steel inserts for fluorescein deposition (lower-left and lower-right), respectively. Figure S2. TEM pictures of ZnO NM110 (nanoparticle suspension prepared in distilled water before nebulization). Figure S3. Left: Particle volume-size distribution of ZnO nanoparticles dispersed in water revealed volume/mass median diameter and geometric standard deviation of 290 nm of 1.44, respectively (dynamic light scattering, suspension concentration: 1 mg/ml). Right: Comparison of the volume-size distributions before and after nebulization (0.5 mg/ml, liquid droplet was collected in an eppendorf tube upon nebulization and subsequently measured by DLS). Volume/mass- median diameter: 256.7 (before) and 272.7 (after) nm. Figure S4. QCM stability (1 Hz data) at zero-point level (unloaded QCM of Cloud 6) under thermal equilibrium (ca. 37 °C). Three repeated measurements were conducted (T1, T2, T3) for 1 h (3,600 data points each). If the zero point of the QCM is set by the operator just prior to the experiment at an “arbitrarily” selected data point, this can [file 12989_2020_376_MOESM1_ESM.docx]

**Supplemental Information (SI)**

**For**

**Quartz crystal microbalances (QCM) are suitable for real-time dosimetry in nanotoxicological studies using VITROCELL**®**Cloud cell exposure systems**

Yaobo Ding^1,2^, Patrick Weindl^1,2,3^, Paula Mayer^1,2^, Tobias Krebs^3^ and Otmar Schmid^1,2^

^1^Institute of Lung Biology and Disease, Helmholtz ZentrumMünchen, 85764 Neuherberg, Germany
^2^Comprehensive Pneumology Center - Member of the German Center for Lung Research (DZL),
81377 Munich, Germany

^3^VITROCELL Systems GmbH, 79183 Waldkirch, Germany

| Parameters | Cloud 6 | Cloud 12 |
| --- | --- | --- |
| Pre-filled DPBS volume in insert, ml | 0.6 | N.A. |
| DPBS volume for QCM washing, ml | 0.6 | N.A. |
| Stainless steel Insert deposition area, cm^2^ | 4.9 | N.A. |
| QCM deposition area, cm^2^ | 3.8 | 1.04 |
| Surface area of chamber bottom, cm^2^ | 143.6 | 136.5 |
| Nebulized volume, µl | 300 | N.A. |

Table S1 Parameters for calculation of deposition efficiency (Cloud 6)

|  | Dose 1 | Dose 2 | Dose 3 | Dose 4 | Dose 5 |
| --- | --- | --- | --- | --- | --- |
| Target dose, cm^2^/cm^2^ | 0.1 | 0.3 | 0.5 | 0.9 | 1.5 |
| Particle concentration, mg/ml | 2 | 2 | 2 | 2 | 4 |
| Nebulized suspension volume, µl | 120 | 359 | 748 | 1077 | 897 |
| Insert delivered volume, µl | 1.75 | 5.24 | 10.91 | 15.75 | 13.08 |
| SA_Chamber_(Cloud 6) = 143.6 cm^2^, BET_particle_ = 12 m^2^/g, DF = 0.5 | | | | | |

Table S2 Target doses and associated conditions of suspension and nebulization to be adopted during cell exposure

QCM Accuracy: F-test was done to determine whether the QCM-value is able to predict the fluorescein-measured deposition. The detailed results are presented in the Table S3.

| Regression | Cloud 6 | Cloud 12 |
| --- | --- | --- |
| a | 1.0339 | 0.9620 |
| se a | 0.0092 | 0.0095 |
| R² | 0.9997 | 0.9997 |
| b | -0.29 | -68.50 |
| se b | 50.9 | 47.07 |
| se y | 90.22 | 73.28 |
| Degrees of freedom | 3 | 3 |
| F | 12625.3 | 10044.5 |
| Fcrit | 9.276628153 | 9.27662815 |
| Accuracy | 3.40% | 3.79% |
| Error | 0.92% | 0.96% |
| Linearity | 0.9998 | 0.9997 |
| F-test | True | True |

Table S3 Results of F-test on correlations between QCM measurements and fluorescein depositions for both Cloud systems

| QCM_ref_ (ng/cm²) | 206.7 | 750 | 1149 | 2757 | 11647 |
| --- | --- | --- | --- | --- | --- |
| Mean SD (ng/cm²) | 46.2 | 42.63 | 45.55 | 41.59 | 33.23 |
| Detection limit, (ng/cm²) | 138.5 | 127.9 | 136.65 | 124.77 | 99.68 |
| Precision | 22.3% | 5.68% | 3.96% | 1.51% | 0.29% |
| QCM_ref-indirect_^*^(ng/cm²) | 10922 | 16383 | 21844 | 43688 | 65533 |
| Mean SD (ng/cm²) | 141.46 | 52.07 | 87.62 | 105.98 | 53.58 |
| Detection limit, (ng/cm²) | 424.37 | 156.21 | 262.86 | 317.94 | 160.75 |
| Precision | 1.30% | 0.32% | 0.40% | 0.24% | 0.08% |
| ^*^For high doses the reference value was calculated based on the known deposition factor of 64.7% (not from direct fluorescein measurement as for values below ca. 11,000 ng/cm^2^); S.D. – standard deviation | | | | | |

Table S4 Electronic noise induced precision (relative standard deviation (SD)) and lower detection limit (3 SD) of the VITROCELL® Cloud 6 for 1 Hz QCM data (for 60 s averaged QCM signal) for fluorescein-spiked and pure salt nebulizations

| QCM_ref_ (ng/cm²) | 194 | 972 | 2086 | 4735 | 9632 |
| --- | --- | --- | --- | --- | --- |
| Mean SD (ng/cm²) | 3.58 | 4.75 | 6.63 | 5.01 | 6.07 |
| Detection limit, (ng/cm²) | 10.74 | 14.26 | 19.88 | 15.03 | 18.21 |
| Precision | 1.84% | 0.49% | 0.32% | 0.11% | 0.06% |
| QCM_ref-indirect_*(ng/cm²) | 5788 | 11576 | 23152 | 46304 | 69457 |
| Mean SD (ng/cm²) | 8.48 | 7.66 | 4.85 | 12.53 | 33.99 |
| Detection limit, (ng/cm²) | 25.44 | 22.98 | 14.55 | 37.60 | 101.96 |
| Precision | 0.15% | 0.07% | 0.02% | 0.03% | 0.05% |
| * For high doses the reference value was calculated based on the known deposition factor of62.1% (not a direct fluorescein measurement); S.D. – standard deviation | | | | | |

Table S5 Electronic noise induced precision (relative standard deviation (SD)) and lower detection limit (3 SD) of the VITROCELL® Cloud 12 for 1 Hz QCM data (for 30 s averaged QCM signal) for fluorescein-spiked and pure salt

As seen from Table S4 the electronic noise induced detection limit (based on standard deviation of 1 Hz QCM signal during phase III; Figure 2) at the lowest achievable dose level for Cloud 6 is 138.5 ng/cm^2^. Averaging over 60 s reduces the electronic noise induced detection limit to ca. 10% of the overall detection limit of 170 ng/cm^2^. Similarly, the electronic noise induced detection limit at the lowest dose level for Cloud 12 is 10.74 ng/cm^2^ (Table S5) which is already less than 10% of overall detection limit of 169 ng/cm^2^. Thus, 30 s of average time is sufficient for operation of the QCM of the Cloud 12.


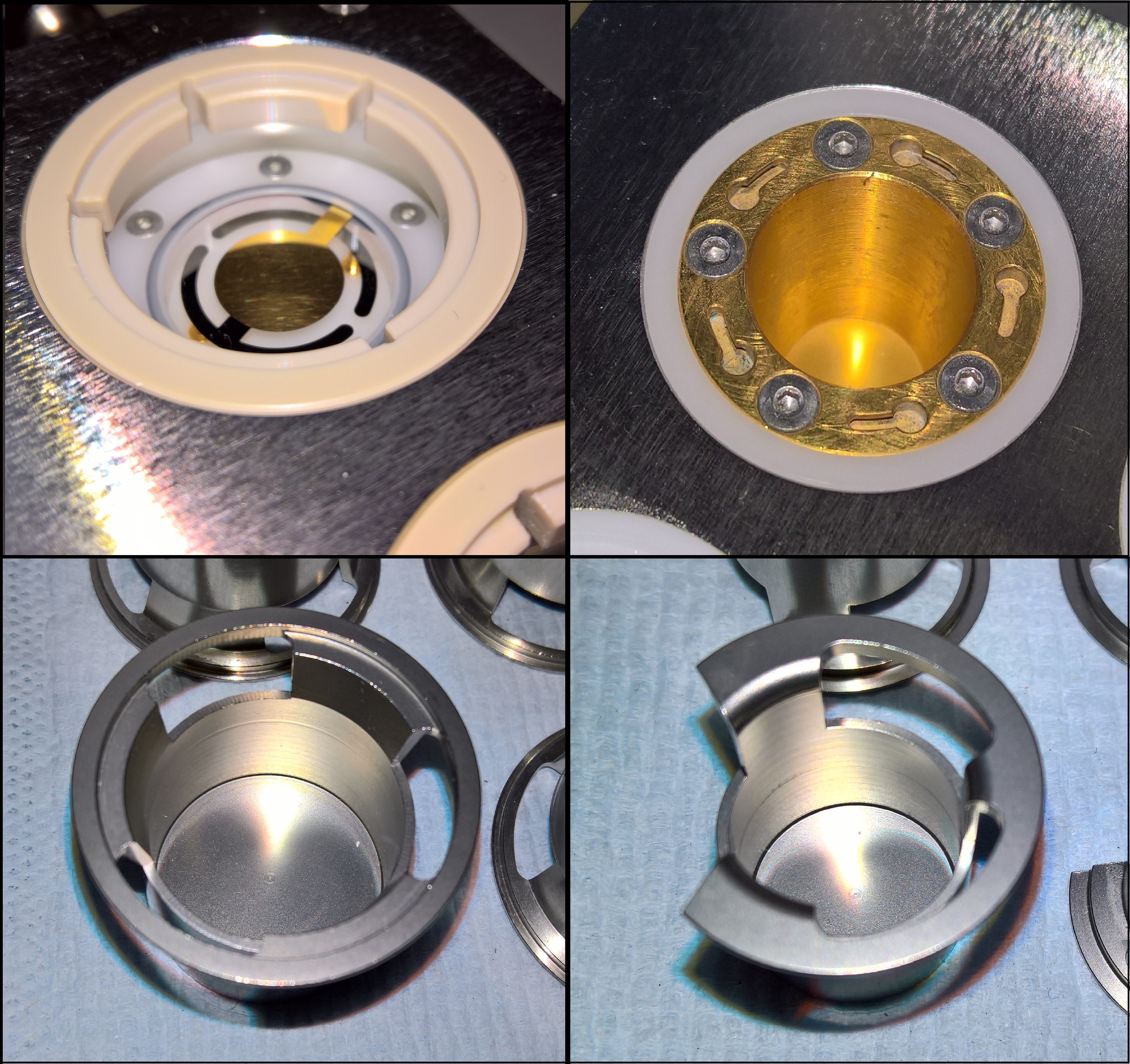


Figure S1 – Photos of the QCM setups in the VITROCELL® Cloud 6 (upper-left) and 12 (upper-right) and corresponding 6-well and 12-well stainless steel inserts for fluorescein deposition (lower-left and lower-right), respectively.


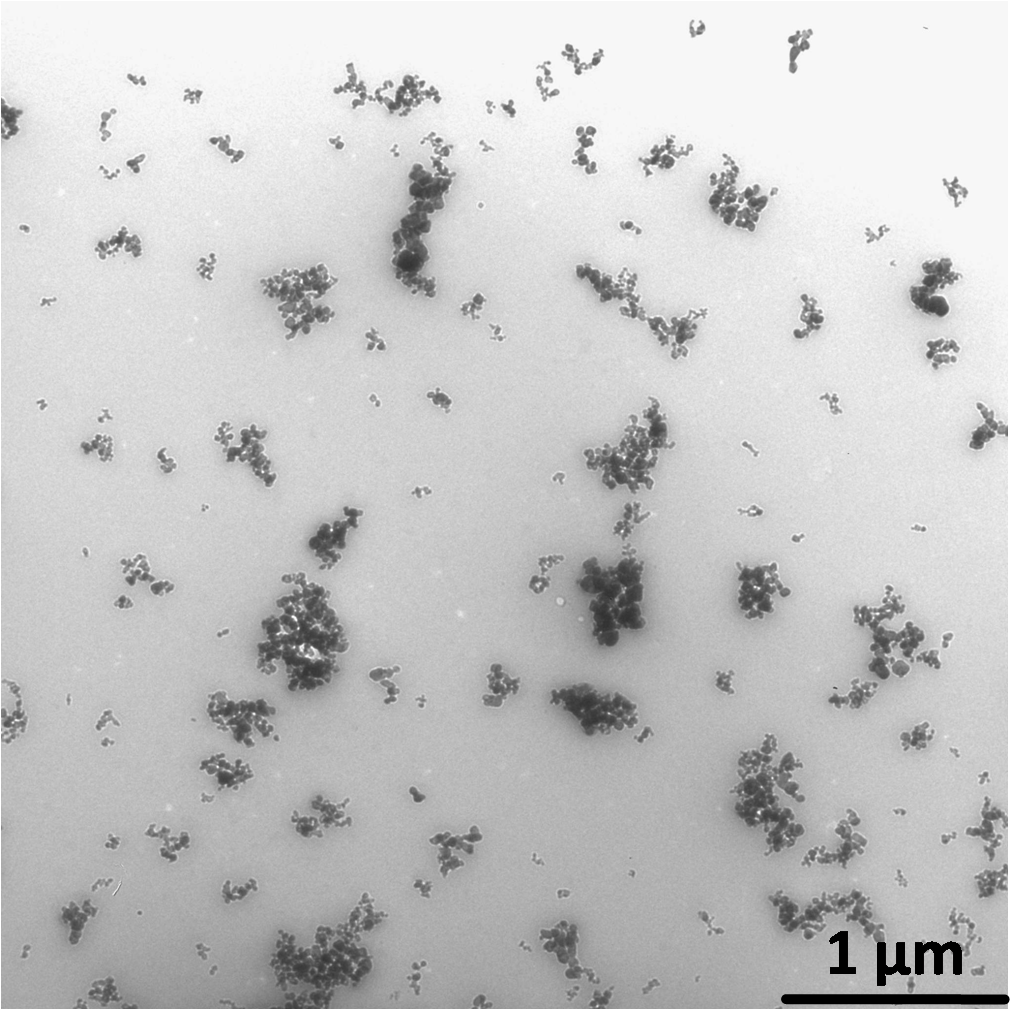

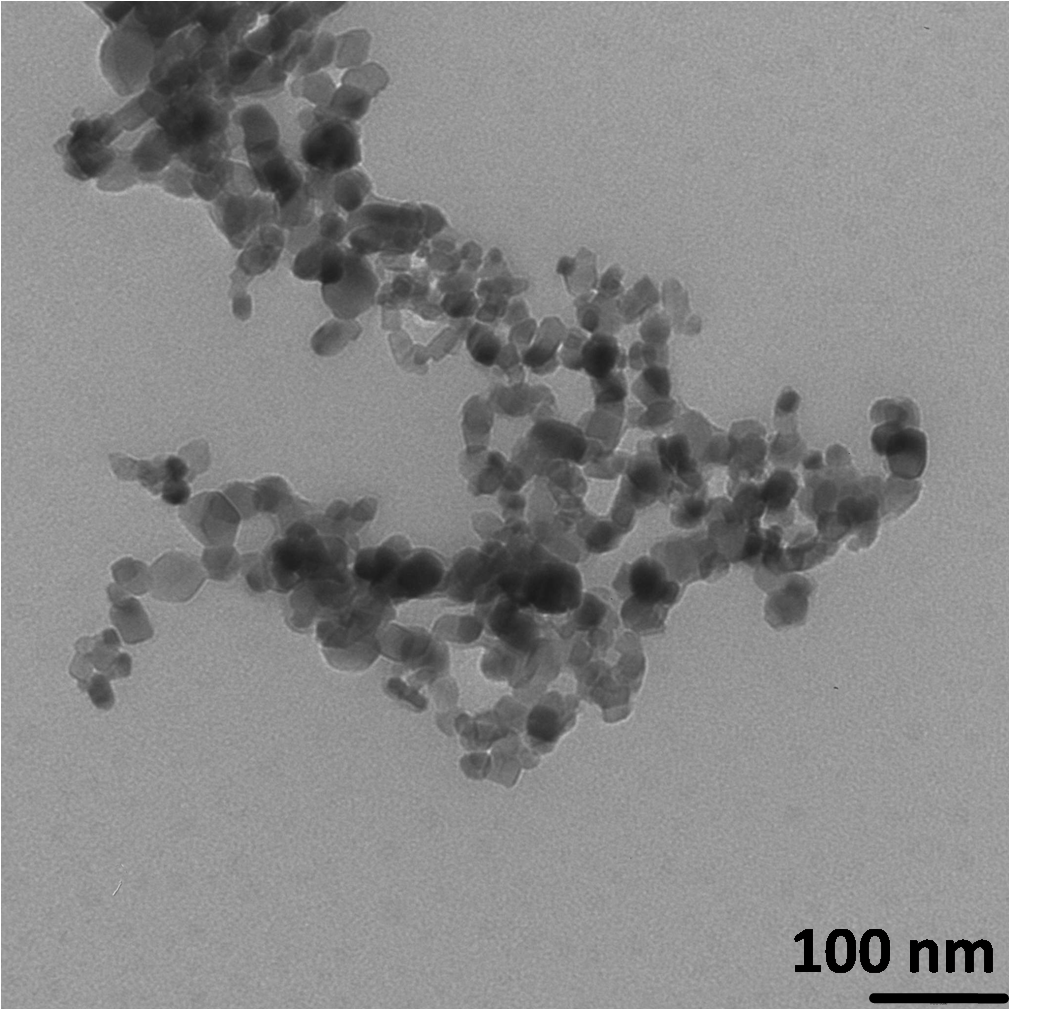


Figure S2 TEM pictures of ZnO NM110 (nanoparticle suspension prepared in distilled water before nebulization)

Figure S3 Left: Particle volume-size distribution of ZnO nanoparticles dispersed in water revealed volume/mass median diameter and geometric standard deviation of 290 nm of 1.44, respectively (dynamic light scattering, suspension concentration: 1 mg/ml). Right: Comparison of the volume-size distributions before and after nebulization (0.5 mg/ml, liquid droplet was collected in an eppendorf tube upon nebulization and subsequently measured by DLS). Volume/mass- median diameter: 256.7 (before) and 272.7 (after) nm.

Figure S4 QCM stability (1 Hz data) at zero-point level (unloaded QCM of Cloud 6) under thermal equilibrium (ca. 37°C). Three repeated measurements were conducted (T1, T2, T3)for 1 hour (3,600 data points each). If the zero point of the QCM is set by the operator just prior to the experiment at an “arbitrarily” selected data point, this can result in a “false” mean zero point level (here between ‑53.4 and +47.3 ng/cm^2^ (Table S6)

| Deposition, ng/cm^2^ | Test 1 | Test 2 | Test 3 |
| --- | --- | --- | --- |
| Average | -53.4 | 47.3 | -23.2 |
| Stand. Dev. (1Hz) | 24.9 | 34.3 | 37.9 |
| Mean Stand. Dev. | 32.4 | | |

Table S6 Numeric evaluation of zero point measurements depicted in Figure S4.

Figure S5 Equivalent to Figure S4, but for the Cloud 12 system with a “false” mean zero point level between 3.5 and 21.8 ng/cm^2^ (Table S7)

| Deposition, ng/cm^2^ | Test 1 | Test 2 | Test 3 |
| --- | --- | --- | --- |
| Average | 21.8 | 3.5 | 7.0 |
| Stand. Dev. | 6.8 | 2.2 | 4.0 |
| Mean Stand. Dev. | 4.3 | | |

Table S7 Numeric evaluation of zero point measurements depicted in Figure S5.
